# Eliminating VEGFA+ tumor-associated neutrophils by antibody-drug conjugates boosts antitumor immunity and potentiates PD-1 immunotherapy in preclinical models of cervical cancer

**DOI:** 10.1038/s41419-025-07402-9

**Published:** 2025-02-19

**Authors:** Shili Yao, Lu Sun, Ye Lu, Xiu Zhu, Rui Xu, Tong Yang, Huarong Tang, Peng Guo, Tao Zhu

**Affiliations:** 1 School of Materials Science and Engineering, Faculty of Medicine, Tianjin University, Tianjin, China; 2 Clinical and Translational Research Center, Hangzhou Institute of Medicine (HIM), Chinese Academy of Sciences, Zhejiang, China; 3 Department of Gynecological Oncology, Zhejiang Cancer Hospital, Hangzhou, China; 4 Department of Pathology, Zhejiang Cancer Hospital, Hangzhou, China; 5 Institute of Molecular Medicine, Hangzhou Institute for Advanced Study (UCAS), Hangzhou, China; 6 Department of Gynecological Radiotherapy, Zhejiang Cancer Hospital, Hangzhou, China

**Keywords:** Targeted therapies, Preclinical research, Cancer microenvironment

## Abstract

Tumor-associated neutrophils (TANs) actively interact with antibody-drug conjugates (ADCs) within the tumor microenvironment (TME), though the detailed mechanisms governing their response to ADC treatment remain to be fully elucidated. Herein, we explored how ICAM1-targeted ADCs affect TAN dynamics in preclinical models of cervical cancer. We discovered that I-DXd, our in-house ADC targeting cervical cancer, effectively eliminates tumor cells through specific antigen recognition while concurrently depleting pro-tumor VEGFA + TANs via Fcγ receptor-mediated phagocytosis. This dual action promotes an immunologically favorable TME. Through comprehensive preclinical studies, we established a foundational understanding of the synergistic benefits of combining I-DXd treatment with PD-1 immune checkpoint inhibition, thereby opening new avenues for therapeutic intervention in advanced cervical cancer.

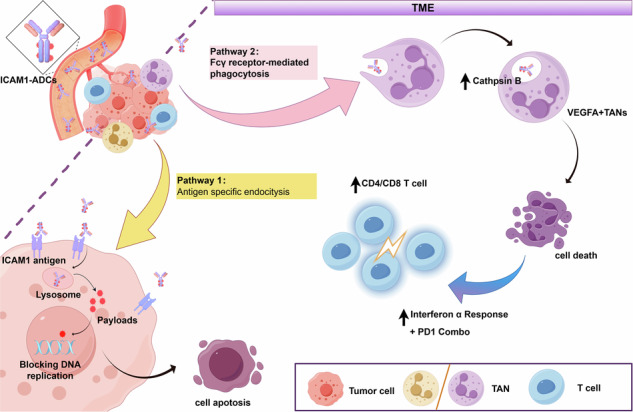

## Introduction

Cervical cancer remains a lethal female cancer accounting for over 0.34 million annual mortalities globally [1, 2]. The clinical outcomes for patients with gynecologic malignancies have not been improved over the past four decades, largely due to the lack of effective treatment modalities [2–5]. Given the lack of second-line therapeutic agents for advanced and metastatic cervical cancer, the 5-year survival rate has plummeted from 92% to 15% and 20% for patients with late-stage cervical cancer [6–8]. Antibody-drug conjugates (ADCs) have emerged as revolutionary targeted therapeutics for various refractory and late-stage solid tumors including cervical and ovarian cancers [9–11]. Tisotumab Vedotin, a tissue-factor-targeted ADC, is the first clinically-approved ADC for advanced cervical cancers. However, in two recent large cohorts of Phase II clinical trials (innovaTV204 and innovaTV207), Tisotumab Vedotin yielded modest therapeutic benefits of overall response rates (ORR) of 24% in patients with advanced cervical cancer and 16% of other solid tumors [12], which is much less effective than Trastuzumab Deruxtecan (ORR: 50%), another HER2-targeted ADC with different linker and payload design, in the DESTINY-PanTumor02 Phase II trial [13]. Therefore, a deeper understanding of the drug mechanism(s) of ADCs is urgently needed to guide the development of novel ADC agents.

Our previous drug mechanism study of ADC chemical structures revealed that the linker and payload combination of Tisotumab Vedotin (MC-VC-PAB-MMAE) conferred significant differences in tumor immune microenvironment in comparison with that of Trastuzumab Deruxtecan (MC-GGFG-DXd), resulting in suboptimal bystander killing effects and therapeutic efficacies in mice [14, 15]. In particular, we found that tumor-associated neutrophils (TANs) were identified as the most affected immune cells by ADCs among innate and adaptive immune cells within the TME. Neutrophils are the most abundant innate immune cells in the human immune system and TANs exhibit functional heterogeneity and plasticity, including pro-tumor and anti-tumor phenotypes in TME [16, 17]. In advanced tumors, TANs commonly are polarized into immunosuppressive phenotypes due to stimuli by tumor cells and TME components [18, 19]. However, the underlying biomechanism(s) of how ADCs affect TANs and subsequent ignited immune responses in TME have yet to be elucidated.

Herein, we identified ICAM1 as an optimal ADC target for cervical cancer and used it to construct two ADCs using the same linker and payload combinations of Tisotumab Vedotin and Trastuzumab Deruxtecan, and comprehensively explored their impacts on TAN elimination and polarization during ADC treatment. These analyses revealed that TANs effectively phagocytose intact ADC macromolecules and facilitated Cathepsin B-mediated linker cleavage, exerting potent bystander killing activity in eliminating VEGFA+ TANs, which in turn, ignites type I interferon (IFN) signaling cascade and increases CD8 + T cell recruitment. These findings suggest that the immunoregulatory functions of ADCs could offer an opportunity to potentiate PD-1 immunotherapy in cervical cancer and other solid tumors.

## Results

### Identification of ICAM1 as a novel ADC target for cervical cancer

**Fig. 1:**
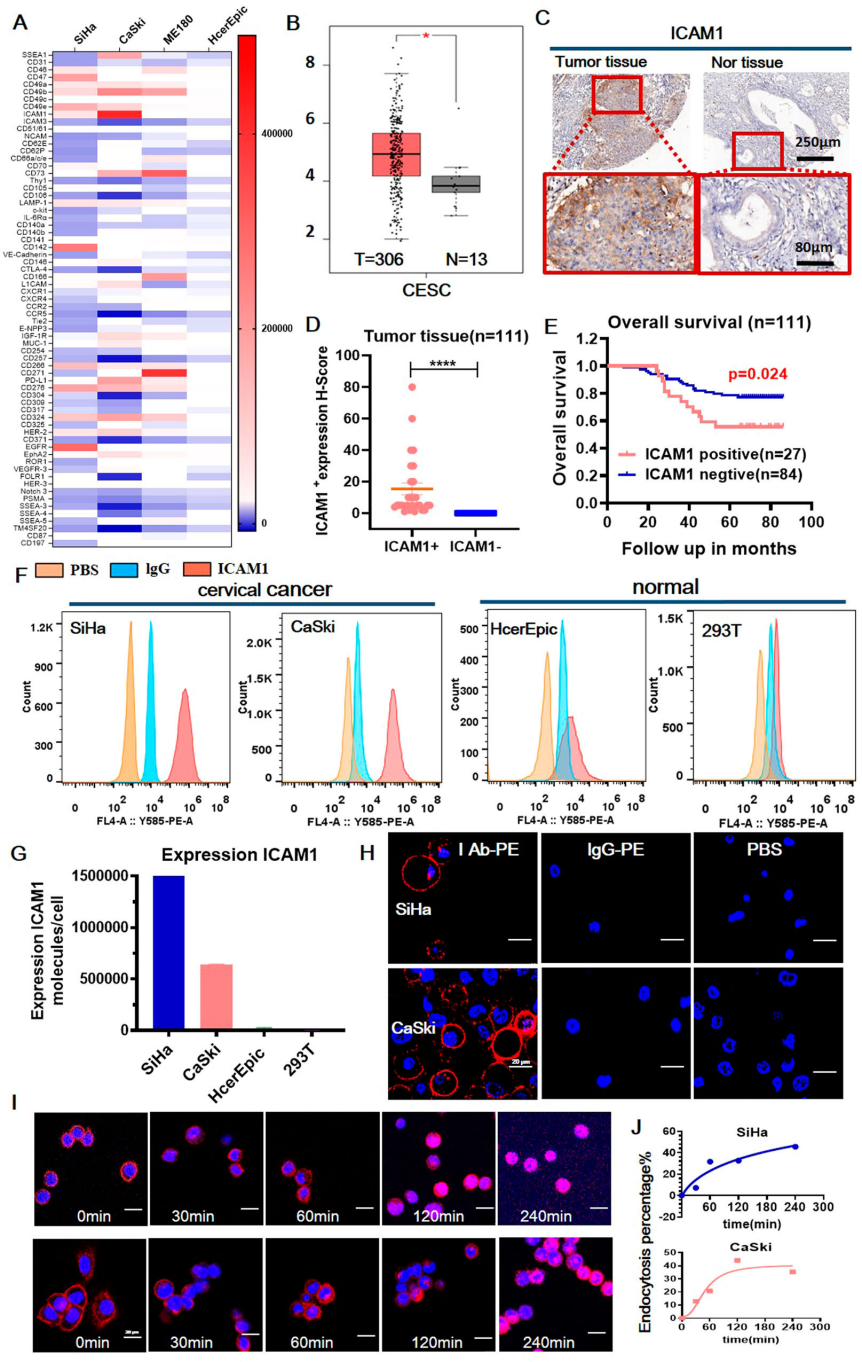
The membrane protein ICAM1 has the potential as an ADC target for cervical cancer. **A** Target ICAM1 is highly expressed in cervical cancer cell lines (SiHa, CaSki, and ME180) by flow cytometry. **B** The GEPIA database indicates significant expression of the ICAM1 gene in patients with cervical cancer. **C** IHC results demonstrate high and specific expression of the ICAM1 in cervical tumor specimens. **D** The expression level of ICAM1 protein in the tumor samples was significantly different. **E** Patients with high ICAM1 expression have an unfavorable prognosis for cervical cancer. **F** Flow cytometry revealed differential expression of ICAM1 in cervical cancer cell lines (SiHa, CaSki,) and normal cells (HcerEpic, 293T). **G** Quantified intensity of ICAM1 protein expressed in cervical cancer cells. **H** Confocal images of plasma membrane ICAM1 expression in cervical cancer cells. **I** Confocal images of the internalization of the ICAM1 antibody by SiHa (upper panel) and CaSki (lower panel) cells at different time points. **J** Quantification of ICAM1 antibody internalization efficiency. The error bars indicate standard error of mean (SEM). Statistical significance in (**D**) was calculated by unpaired *t*-test. Kaplan–Meier survival (**E**) using the log-rank (Mantel–Cox) test for the patients in two groups. ^*^P < 0.05, ^**^P < 0.01, ^***^P < 0.001, ^****^P < 0.0001. IHC images, scale bars, 250 µm and 80 µm, respectively. Immunofluorescence images, scale bars, 20 µm.

We first performed an unbiased and quantitative screening of a library of 67 cell membrane proteins on a panel of three cervical cancer cell lines (SiHa, CaSki, ME180) and normal human cervical epithelial HcerEpic cells. 4 of 67 proteins (ICAM1, CD49b, CD73, and EGFR) were identified as consistently upregulated in all cervical cancer cell lines (Fig. 1a). Notably, ICAM1, an inflammation-related cell membrane protein, showed potential as a specific molecular target candidate for cervical cancer. By querying the Human Protein Atlas database (https://www.proteinatlas.org), CD49b, CD73, and EGFR are highly expressed in normal tissues due to their poor specificity. CD49b was expressed in 71.11% (32/45) of normal tissues, CD73 in 86.05% (37/43), and EGFR in 46.67% (21/45), which may cause more on-target-off-tumor toxicities in contrast to ICAM1 in 19.15% (9/47). Therefore, ICAM1 was selected as the target candidate for further investigation. To validate its clinical relativity, we queried the GEPIA database for ICAM1 expression in a total of 306 patients with cervical cancer and found that the level of ICAM1 mRNA was significantly upregulated in patients with cervical cancer (Fig. 1b).

To validate this finding at the protein level, we performed immunohistochemical (IHC) staining of ICAM1 on a microarray of 111 human cervical tumor tissues including 107 squamous cell carcinoma and 4 adenocarcinoma (Fig. 1c). The IHC staining of ICAM1 expression shows heterogeneous patterns in cervical tumor tissues. Regions with low or absent ICAM1 expression primarily consist of regions of necrosis and non-malignant stromal cells, including fibroblasts, and infiltrating immune cells such as lymphocytes and macrophages, which typically do not express high levels of ICAM1. This contrasts with the areas where tumor cells with high ICAM1 expression are clustered, exhibiting stronger staining. Additionally, the whole tissue section staining (Supplementary Fig. 1) confirms this pattern, showing ICAM1 is predominantly expressed in tumor cell regions while being low or absent in surrounding stromal and non-cancerous areas. Importantly, the ICAM1 positive rate in cervical tumor tissues was determined as 24.32% (27/111). Among them, 26.87% (18/67) were determined as ICAM1-high expression and 21.05% (8/38) as ICAM1-low expression. In comparison, ICAM1 expression is completely absent in 25 paracancerous normal human tissues (Fig. 1d). The IHC staining scoring criteria of ICAM1 are defined as shown in the supplementary information (Supplementary Fig. 2) and the IHC method section. Moreover, high ICAM1 expression was associated with a worse prognosis in patients with cervical cancer (Fig. 1e). We have previously assessed the ICAM1 expression in a cohort of 144 normal human tissues of 20 different organs by IHC showing low or undetectable ICAM1 expression in most organs [20].

We next quantified ICAM1 overexpression in human cervical cancer cells (SiHa and CaSki) by flow cytometry and immunofluorescence staining, which are 80- and 106-fold higher than those of non-neoplastic HcerEpic and 293T cells, respectively (Fig. 1f, g). Moreover, immunofluorescence staining shows that ICAM1 was localized on the plasma membrane of SiHa and CaSki cells (Fig. 1h), which is readily accessible for antibody-based drugs. Given that cell entry is a critical factor affecting the therapeutic efficacy of ADC [21, 22], we evaluated the internalization rate of ICAM1 antibodies in human cervical cancer cells. As shown in Fig. 1i, j, fluorophore phycoerythrin (PE) -conjugated ICAM1 antibodies rapidly entered SiHa and CaSki cells, reaching a plateaued internalization rate of approximately 30% within 120 min. In addition, the cell viability test shows that the ICAM1 antibody did not affect the cell viability during this internalization process (Supplenmentary Fig. 3). Furthermore, the internalized ICAM1 antibodies were observed to colocalize with the lysosomes (LAMP1+, Supplenmentary Fig. 4) of cervical cancer cells, confirming that the internalized antibody traffics to intracellular compartments, validating the internalization process of ICAM1 in these cells. Overall, the above findings strongly support ICAM1 as a promising ADC target for cervical cancer therapy.

### Generation and characterization of ICAM1-ADCs

**Fig. 2:**
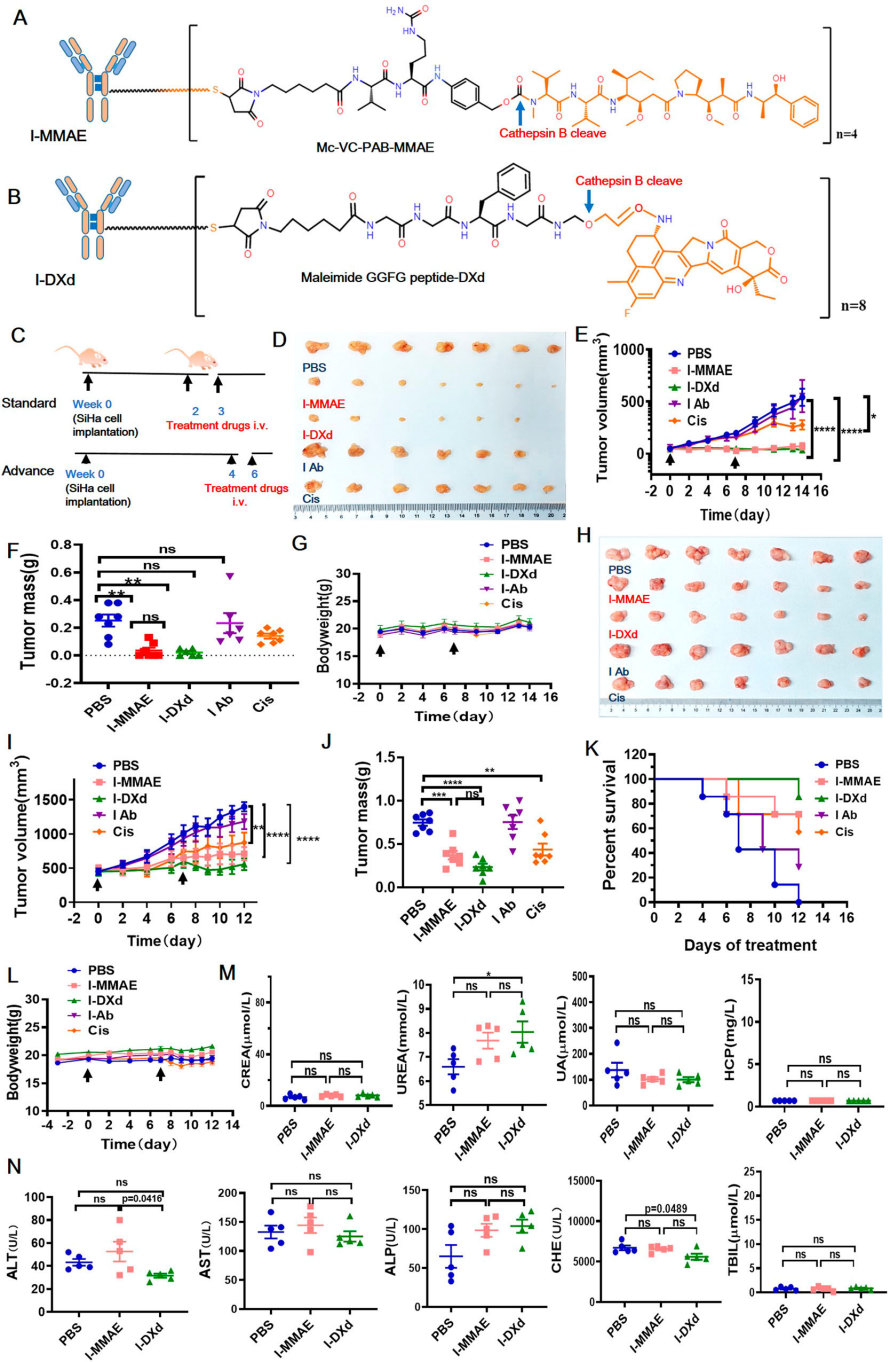
ICAM1 ADCs exhibited favorable antitumor activity and biosafety in vitro and in vivo. **A**, **B** The structure and the molecular formula of two ICAM1 ADCs. **C** Establishing a standard and a late-stage treatment models for cervical cancer in mice. **D** Tumor schematic after standard treatment (*n* = 6 or 7 per group). **E** Tumor growth curves during treatment. **F** Quantification of tumor weight after treatment. **G** Body weight curves of all groups in the standard mouse model. **H** Tumor schematic after late-stage model therapy (*n* = 7 per group). **I** Tumor growth curves of the late-stage model; **J** Quantification of tumor weight of the late-stage model. **K** Comparison of survival curves of the late-stage model; **L** Body weight curves in late-stage therapeutic models. **M** Kidney function index was normal in late-stage therapeutic models (Fig. 2H). **N** Liver function index was normal. The error bars indicate SEM. Statistical significance was calculated by one-way ANOVA with Tukey’s multiple comparison test. Kaplan–Meier survival (**K**) Using the Gehan–Wilcoxon test for the mouse survival data, followed by Bonferroni’s correction for multiple comparisons. ^*^P < 0.05, ^**^P < 0.01, ^***^P < 0.001, ^****^P < 0.0001, ns not significant.

To find the optimal ADC construct for cervical cancer therapy, we compared the antitumor efficacy of two ICAM1-ADCs of different mechanisms of action (MoAs) previously developed by us. The two ICAM1-ADCs were constructed by conjugating ICAM1 monoclonal antibodies with two clinically approved linker and payload combinations from Tisotumab Vedotin and Trastuzumab Deruxtecan, MC-VC-MMAE and MC-GGFG-DXd. The obtained ADCs were termed ICAM1-MMAE (I-MMAE) and ICAM1-DXd (I-DXd) based on their cytotoxic payloads. I-MMAE features a microtubule inhibitor MMAE payload whereas I-DXd uses DNA topoisomerase inhibitor DXd as payload (Fig. 2a, b), which could ablate cervical cancer cells via different MoAs. Both I-MMAE and I-DXd utilized cathepsin B enzyme-cleavable peptide linkers, providing a bystander-killing effect within the TME. The drug-to-antibody ratios (DARs) of I-MMAE and I-DXd were fine-tuned to 4 and 8 (Fig. 2a, b), respectively, in consistency with Tisotumab Vedotin and Trastuzumab Deruxtecan of the same linker and payload combinations [14, 23].

We next determined the in vitro cytotoxicity of two ICAM1-ADC constructs in a panel of cervical cancer (SiHa and CaSki) and non-neoplastic (HcerEpic and 293T) cell lines (Supplementary Fig. 5). The half maximum inhibitory concentration (IC50) of I-MMAE was determined as 0.1198 µg/ml (SiHa) and 1.149 µg/ml (CaSki), respectively. For I-DXd with a less cytotoxic payload DXd, its IC50 was approximately 10 µg/ml for SiHa and CaSki cells. Notably, both ICAM1-ADC constructs did not induce any cytotoxicity on normal human cervical cells (HcerEpic). Cisplatin (Cis) and Paclitaxel (Pac) are two of the conventional first-line chemo drugs for cervical cancer, which were used as positive controls. As shown in Fig. S5, Cis and Pac ubiquitously ablate both cervical cancer and non-neoplastic cells without any tumor-selectivity. These in vitro efficacy results support that two ICAM1-ADCs are potent in cervical cancer cells with high tumor selectivity, warranting further investigation in the in vivo cervical tumor models.

### ICAM1-ADCs potently suppress cervical tumors in vivo

Given the lack of second-line therapeutic agents for advanced and metastatic cervical cancer, the five-year survival rate has plummeted from 92% to 15% and 20% for patients with late-stage cervical cancer [6–8]. Thus, this study evaluated the therapeutic potential of ICAM1-ADCs in both standard and late-stage cervical tumor models. ICAM1-positive SiHa cells were utilized to establish the standard and late-stage models of human cervical tumors in nude mice (Fig. 2c).

In the standard setting, the established protocol entailed administering I-MMAE and I-DXd through the caudal vein once tumor volume reached 50–80 mm^3^. The first-line chemotherapy drug Cis was utilized as a positive control, while ICAM1 antibody (I Ab) and PBS served as the negative controls. The recommended dosage [15, 24] for I-Dxd, I-MMAE, and Cis is 5 mg/kg, administered twice, on day 0 and day 7, respectively (Fig. 2c). After treatment, the tumor in the control group treated with PBS increased linearly while both I-MMAE and I-DXd groups showed a significant reduction in cervical tumor growth (Fig. 2d, c). Notably, there was no significant difference between the efficacies of the two ADCs in the standard setting (Fig. 2c). In comparison, Cis treatment only showed a moderate inhibitory effect on cervical tumors. Quantified terminal tumor weights confirmed that the antitumor effects of two ICAM1 ADCs are significantly more potent than Cis (44.32%) at the same dose with their tumor inhibition rates determined as 85.80% (I-MMAE) and 91.38% (I-DXd), noting that ICAM1 antibody alone had no obvious inhibitory effect on the cervical tumor (Fig. 2f).

In the late-stage setting [25], ICAM1 ADC treatment was initiated when the tumor volume reached approximately 500 mm^3^. Consistently, both I-MMAE and I-DXd showed potent tumor inhibitory activity in comparison with other control groups, with I-DXd exhibiting the strongest antitumor effect (Fig. 2). Their tumor inhibition rates in the late-stage setting were determined as 50.10% (I-MMAE) and 68.64% (I-DXd), substantially more potent than 41.49% of the Cis group (Fig. 2j). We further evaluated animal survival by Kaplan-Mier analysis, and in comparison with the PBS group, two ICAM1-ADC groups significantly improved their prognosis, which was consistent with their antitumor effects (Fig. 2k). While we recognize that this categorization of standard and late-stage cervical tumor models does not capture inherent molecular differences in cervical cancer subtypes, it allows us to assess the therapeutic efficacy of ADCs at varying tumor burdens, which is relevant for understanding treatment response in clinical settings with different tumor sizes. Collectively, the I-DXd construct exhibits consistently superior antitumor activity with improved prognosis in both standard and late-stage models, which is recommended for further cervical cancer therapy studies.

### ICAM1 ADCs are safe and well-tolerated in mice

We assessed the toxicity of I-MMAE and I-DXd in multiple human cervical cell line-derived xenograft (CDX) models via blood biochemistry. First, we observed no significant bodyweight changes upon ICAM1 ADC treatment in both standard and late-stage settings of SiHa tumors (Fig. 2g, i). We further analyzed a panel of serum biomarkers related to liver and renal functions and immunogenicity [26, 27]. As shown in Fig. 2n, I-MMAE and I-DXd treatments did not alter the levels of ALT (glutamic pyruvic transaminase), AST (glutamic oxaloacetic transaminase), ALP (alkaline phosphatase), CHE (cholinesterase), and TBIL (total bilirubin) related to liver functions. Similar trends were also observed in renal function and immunogenicity biomarkers including CREA (creatinine), UREA (urine urea nitrogen), UA (uric acid), and HCP (hypersensitive C-reactive protein) (Fig. 2m). Collectively, the above findings support that the I-MMAE and I-DXd single agents are safe and well tolerated in both standard and late-stage mice settings.

### I-DXd downregulates neutrophil activation in cervical TME and ablates pro-tumor TANs

We previously reported that TANs are the most affected immune cells in TME by Trastuzumab Deruxtecan, a clinical HER2-targeted ADC with the same linker and payload combination with I-DXd [15]. In this study, we specifically investigated the detailed immunoregulatory functions of I-DXd on TANs in cervical TME. Neutrophils are the most numerous innate immune cells in TME and have the dual nature of pro-tumor and anti-tumor during tumor progression [16, 17].

**Fig. 3:**
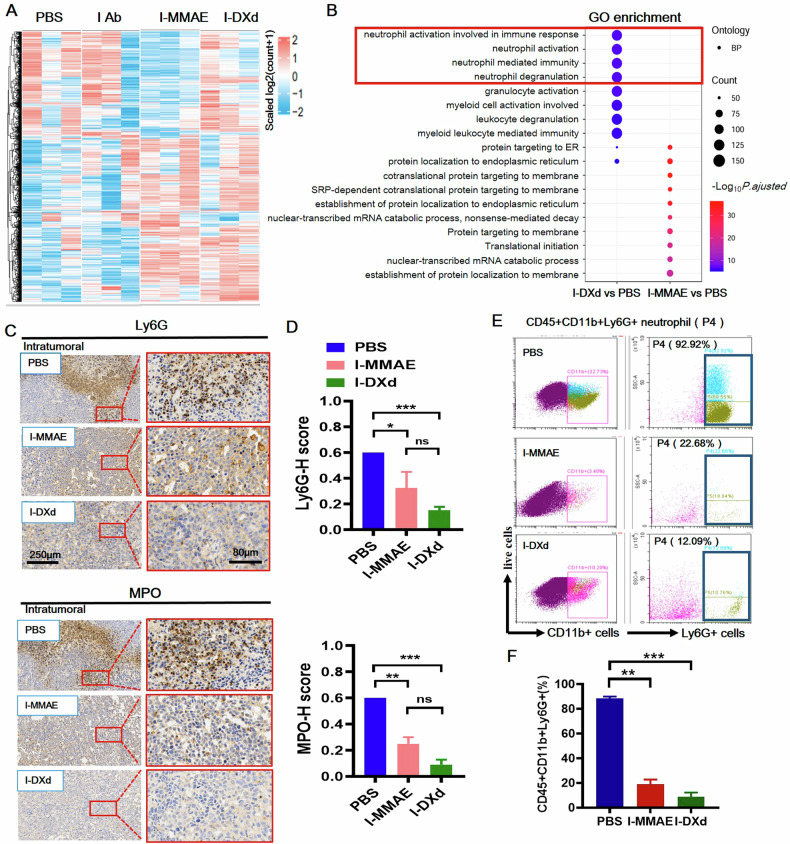
TANs were down-regulated in the mouse model of late-stage cervical cancer after ICAM1 ADC treatment. **A** Comparing the top 1000 differentially expressed genes in different treatments. **B** Downregulation of neutrophil activation processes was observed after I-DXD treatment. **C** IHC schematic of Ly6G and MPO in the late-stage cervical cancer mouse model (*n* = 2 or 3 per group). **D** H-score quantization measures IHC results (**C**). **E** Flow cytometric showed a significant decrease of TAN (P4 gating for CD45 + CD11b + Ly6G + ) after ICAM1-ADCs treatment (*n* = 2 per group). **F** Quantification of FACS results (e-graph). The error bars indicate SEM. Statistical significance was calculated by one-way ANOVA with Tukey’s multiple comparison test. ^*^P < 0.05, ^**^P < 0.01, ^***^P < 0.001, ^****^P < 0.0001, ns not significant. IHC images, scale bars, 250 µm and 80 µm, respectively.

We first performed the whole transcriptomic analysis of ICAM1-ADC treated cervical tumors from the late-stage tumor models via RNA-sequencing (RNA-seq). Figure 3a shows the top 1000 differentially expressed genes (DEGs) of PBS, ICAM1 antibody, I-MMAE, and I-DXd groups, and the I-DXd group exhibited a significantly changed phenotype in comparison with other groups. Gene Ontology (GO) enrichment analysis revealed that I-DXd significantly downregulated four pathways associated with neutrophil activation different from I-MMAE (Fig. 3b), indicating that the neutrophil activation process was suppressed in cervical TME after I-DXd treatment. Next, we performed an IHC staining on the ICAM1 ADC-treated cervical tumors harvested from the late-stage model. TANs were labeled by Ly6G and pro-tumor subsets of TANs were identified by MPO, an established biomarker for tumor-promoting neutrophils [28, 29]. IHC staining results (Fig. 3c) showed that I-MMAE and I-DXd potently eliminated tumor-infiltrating TANs in TME at both total TANs (Ly6G+) and pro-tumor TANs (MPO+) levels, and I-DXd reached a superior TAN elimination rate than I-MMAE, as high as 75% for total TANs and 85% for pro-tumor TANs, respectively (Fig. 3d). We validated this critical finding in an independent flow cytometry assay (Fig. 3e) and found that CD45 + CD11b + Ly6G + TANs accounted for 92.92% of the CD11b+ myeloid cells in the PBS group, while it was significantly reduced to 22.68% and 12.09% of the myeloid cells in the I-MMAE and I-DXd groups, respectively (Fig. 3f). These findings strongly support that I-MMAE and I-DXd effectively eliminate pro-tumor TANs (Ly6G + MPO + TANs) in cervical TME.

In addition, the heterogeneous staining signals of Ly6G and MPO in the PBS-treated group reflect the variable distribution of neutrophils within the tumor microenvironment (Fig. 3c and Supplementary Fig. 6). This pattern indicates that neutrophils are not uniformly distributed across the tumor tissue, with certain regions showing a higher density of Ly6G+ and MPO+ cells. This variability is commonly observed in tumors, where factors contributing to this distribution pattern include tumor architecture, hypoxia, and intra-tumoral chemokine gradients within the tumor microenvironment [16, 30]. In areas with higher neutrophil infiltration, Ly6G, and MPO staining intensities are stronger, likely due to the recruitment of neutrophils in response to local tumor cues. Conversely, regions with lower staining indicate areas where neutrophil infiltration is minimal or absent. Such heterogeneous distribution aligns with the dynamic and complex nature of the tumor microenvironment, where immune cell populations often exhibit spatial variability.

### I-DXd effectively ablates VEGFA + TANs and boosts antitumor immunity

**Fig. 4:**
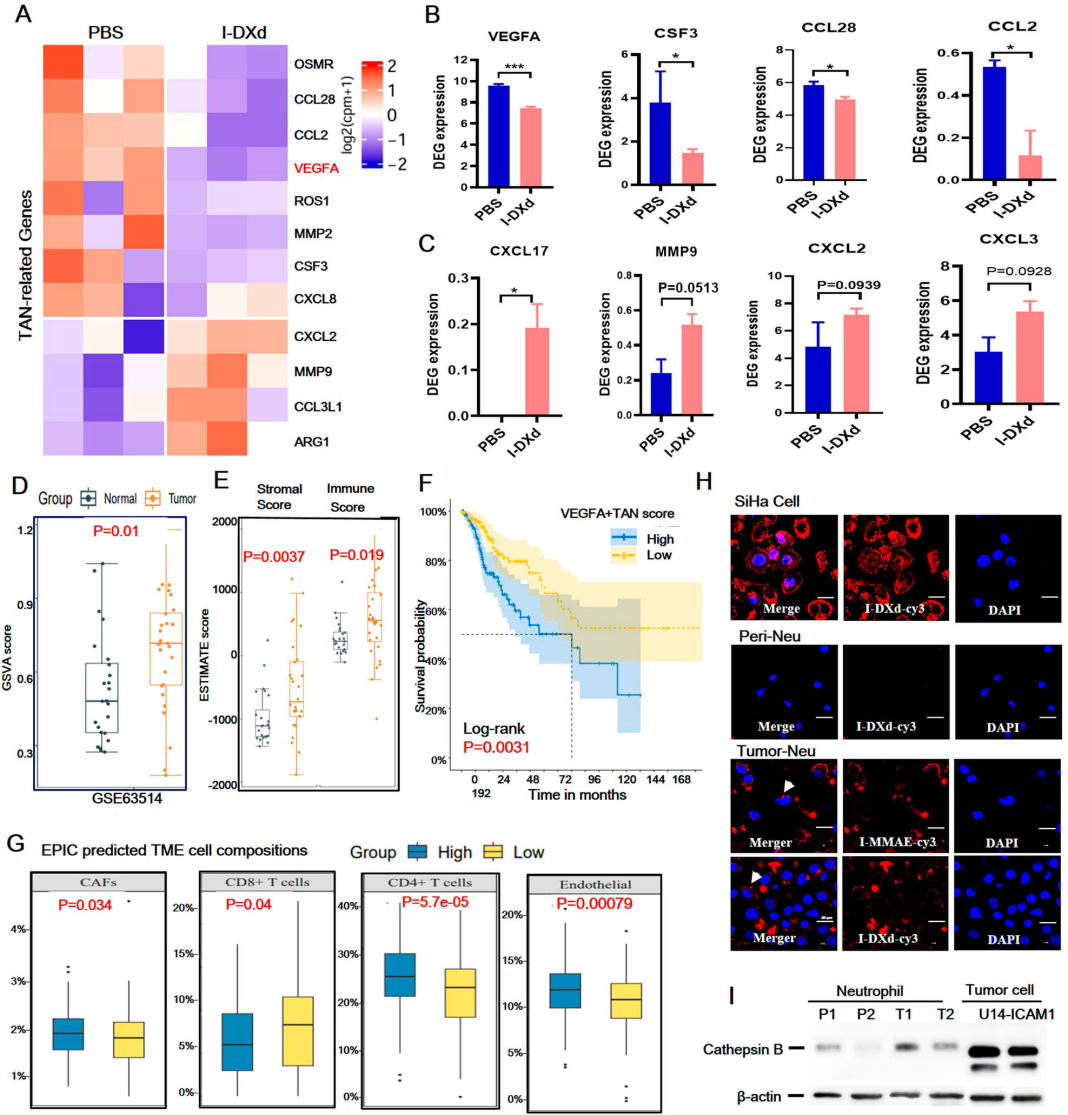
ICAM1-ADCs ablate pro-tumor TANs and boost antitumor immunity. **A** Differential expression of TAN-related genes. **B** Protumoral genes related to TAN are significantly downregulated (*n* = 3 per group). **C** Antitumoral genes related to TAN are significantly upregulated. **D**, **E** VEGFA+TANs were significantly enriched in tumors, stromal, and immune cells in the clinical database GSE63514. **F** Kaplan–Meier analysis of high- and low-TANs GSVA score in TCGA database. **G** EPIC predicted TME cell composition between high and low TAN-related GSVA score groups in the TCGA database. **H** Confocal imaging demonstrated that ICAM1-ADCs were significantly internalized by tumor cells and TANs (white arrows), but not by peripheral blood neutrophils. **I** Western blot analysis results indicate that cathepsin B expression was significantly higher in TAN (T1, T2) than in peripheral blood neutrophils(P1, P2). The error bars indicate SEM. Statistical significance in (**B**–**D** and **G**) was calculated by unpaired *t*-test. Kaplan–Meier survival (**F**) using the log-rank (Mantel–Cox) test for the patients in two groups. ^*^P < 0.05, ^**^P < 0.01, ^***^P < 0.001, ^****^P < 0.0001. Immunofluorescence images, scale bars, 20 µm.

Previous studies have identified 20 cytokine biomarkers associated with TAN functionality in the TME [30]. By comparing the gene scores of differentially expressed genes between I-DXd and PBS control groups in TAN-related gene sets, we identified 12 significant target genes, among which 8 genes were significantly downregulated and 4 genes upregulated (Fig. 4a). Some studies defined VEGFA, CCL2, CCL28, and CSF3, which are related to TAN, as protumor genes in TME, whereas ARG1, MMP9, and CXCL2 were defined as antitumor genes [31, 32]. We further performed a more detailed TAN-associated gene analysis and found that I-DXd significantly downregulates protumor TAN genes (e.g., VEGFA, CCL2, CCL28, and CSF3) (Fig. 4b) and upregulated antitumor TAN genes (e.g., ARG1, MMP9, and CXCL2) (Fig. 4c). Thus, we can reasonably hypothesize that TANs with these 12 significant TAN-related target genes are defined as VEGFA + TANs, which appear to be an immunosuppressive population in the advanced cervical cancer TME.

To assess the clinical relevance of VEGFA+ TANs, gene set variation analysis (GSVA) was employed, demonstrating significant enrichment of these TANs in tumor samples over normal tissues within the GSE63514 database (Fig. 4d, e). Furthermore, a high GSVA score for VEGFA+ TANs correlated with a poorer clinical prognosis in cervical cancer patients according to the TCGA database (Fig. 4f). In correlation, a recent TAN profiling study also reported that the VEGFA + SPP1+ subset was associated with worse clinical outcomes in 225 samples from 145 patients of 17 solid tumors including cervical cancer [33, 34]. We next used EPIC (https://gfellerlab.shinyapps.io/EPIC_1-1), a tool for predicting TME cell composition, to investigate changes in the proportion of immune cells in the TME of cancer patients between high and low TAN-related GSVA score groups in the TCGA database. Differences in immune cell proportions were observed between patients with high and low TAN-related GSVA scores, suggesting a link between lower scores and improved clinical outcomes, possibly due to enhanced CD8 + T cell activation (Fig. 4g). These findings further support the concept that VEGFA+TANs, as a whole, exhibit a tumor-promoting function of immunosuppression in the cervical cancer clinical database, which is reversed or eliminated by I-DXd treatment.

We next investigated the interaction between I-DXd and TANs. Given TANs are innate immune cells with phagocytic functions of foreign microparticles including bacteria and viruses [17], we isolated mouse TANs from human cervical tumors (SiHa) and co-incubated them with fluorophore Cy5.5-labeled I-DXd and I-MMAE in comparison with circulating neutrophils (quiescent) derived from mouse peripheral blood. As a result, mouse TANs showed effective phagocytosis of both ICAM1-ADC constructs, whereas quiescent neutrophils did not show obvious phagocytosis of these ADCs (Fig. 4h). Because the ICAM1 antibody of these ADC constructs only binds human antigens without mouse reactivity, the TAN phagocytosis of ICAM1-ADCs is independent of ICAM1 antigen but dependent on the FcγR engagement [35–37]. Strikingly, our immunoblot results (Fig. 4i and Supplementary Fig. 7) revealed that mouse TANs (T1 and T2) also express high levels of Cathepsin B, the major protease cleaving peptide linkers of ICAM1-ADCs, in comparison with peripheral neutrophils (P1 and P2), indicating that TANs could cleave intact ICAM1-ADCs into small molecular payloads and subsequently exert the bystander killing effect, as well as antigen-positive tumor cells in TME, leading to enhanced an amplified antitumor activity of ICAM1-ADCs. Collectively, these findings elicit that ICAM1-ADCs effectively ablate protumor VEGFA+ TANs in TME through antigen-independent phagocytosis and TAN-mediated bystander-killing effects.

### I-DXd potentiates PD-1 immunotherapy via type I IFN responses

**Fig. 5:**
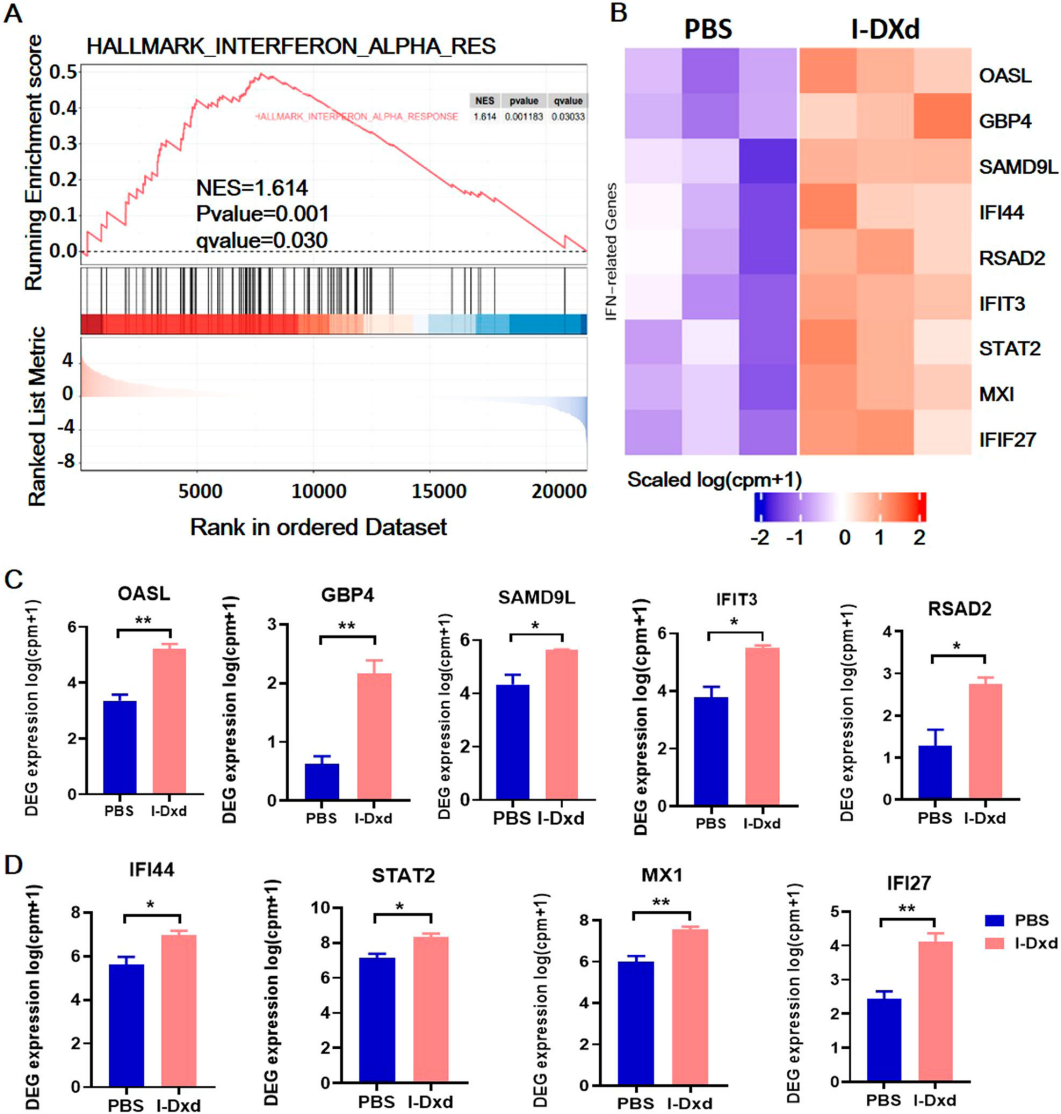
I-DXd activating type I IFN responses and improving the TME. **A** GSEA results showed that the interferon-related pathway IFN alpha was activated. **B** Differential expression of IFN-related genes (*n* = 9) is observed. **C**, **D** IFN-related genes are significantly upregulated; the error bars indicate SEM. Statistical significance in (**C**, **D**) was calculated by unpaired *t*-test for the mice in two groups. ^*^P < 0.05, ^**^P < 0.01, ^***^P < 0.001, ^****^P < 0.0001.

We investigated the immunoregulatory functions of ADC-mediated TAN elimination on tumor immune microenvironment. Based on our transcriptome analysis on I-DXd-treated tumors, the GSEA map shows an enriched interferon (IFN) alpha-related pathway, an established type I IFN immune response (Fig. 5a). Upregulation of IFN signaling in the TME is closely associated with activation of adaptive immunity and antitumor immune stimulation [38–40]. In Fig. 5b, our DEG analysis of IFN signal pathway-related genes showed significant upregulation of a panel of immunoreactive genes including OASL, GBP4, SAMD9L, IFIT3, RSAD2, IFI44, STAT2, MX1, and IFIF27 upon I-DXd treatment (Fig. 5c, d), indicating that I-DXd effectively ameliorates tumor immune microenvironment by activating type I IFN responses. Since type I IFN signaling has an essential role in the full-blown efficacy of many immunotherapy agents, especially PD1 immune checkpoint blockade therapy [41], we postulated that I-DXd may potentiate PD1 immunotherapy by boosting antitumor immunity through TAN elimination.

Additionally, to confirm that immune activation (such as the IFN signaling pathway) is indeed induced by I-DXd treatment rather than ICAM1 antibody, we analyzed the potential signaling transductions in human cervical tumors by ICAM1 neutralizing antibody using RNA transcriptomic analyses. GO analysis (Supplementary Fig. 8) demonstrates the top 20 up- and down-regulated signaling pathways initiated by ICAM1 neutralizing antibody treatment. The regulation of cellular response to the hypoxia pathway was found as the most significantly downregulated signaling pathway upon ICAM1 neutralizing antibody treatment, but further studies are needed to characterize the specific pathways activated in cervical cancer cells. Intriguingly, others have recently reported that ICAM1 neutralizing antibodies effectively inhibit non-small cell lung tumor growth by activating Caspases 3 and 9 and inhibiting AKT and ERK1/2 pathways [42]. In addition, ICAM1 neutralizing antibodies have also been found to inhibit SRC and STAT3 pathways and inhibit colon tumor metastasis and angiogenesis [43].

More importantly, we also examined the I-MMAE on the phenotypes of neutrophils in the same TAN- and IFN-related gene sets (Supplementary Fig. 9). Compared to the I-DXd treatment, the protumoral genes related to TAN are slightly downregulated in the I-MMAE treatment, especially VEGFA, CSF3, CCL28, and CCL2 (Supplementary Fig. 9a, b). Although the panel of immunoreactive genes (IFN-related genes) is slightly upregulated by I-MMAE treatment (Supplementary Fig. 9c, d), I-DXd is more effective in activating neutrophil-related immune responses than I-MMAE as observed in Fig. 3b. Subsequently, we focused on the effects of I-DXd and its combo therapy on immune activation due to its unique mechanism and promising preclinical results.

### I-DXd combined with PD1 antibody induces CD8 + T cell recruitment and enhances the anti-tumor effect

**Fig. 6:**
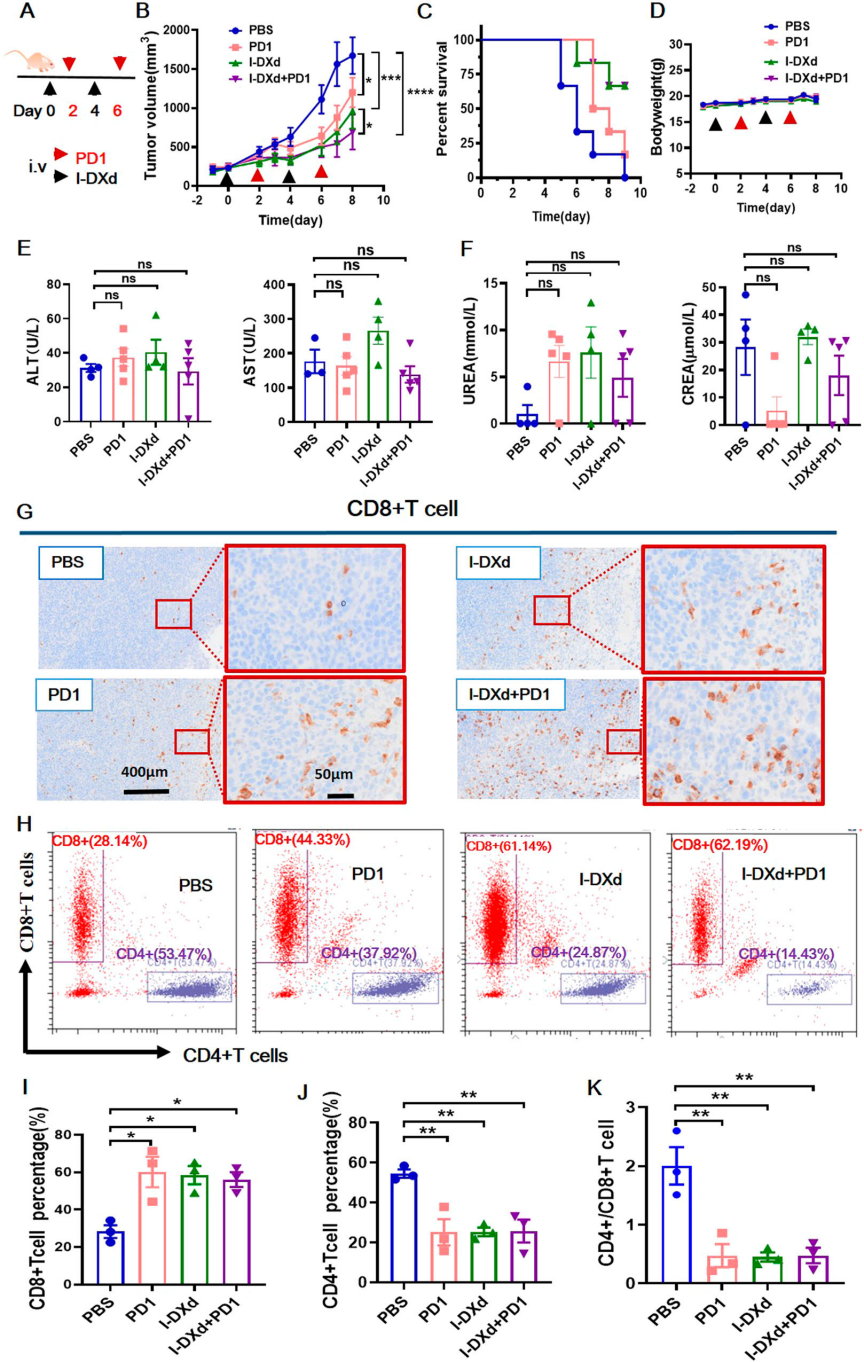
I-DXd combined with PD1 antibody induces CD8 + T cell recruitment and enhances the anti-tumor effect. **A** Establishing a combo treatment model in immunocompetent Balb/c mice (*n* = 6 per group). **B** Tumor growth curves in immunocompetent mouse model. **C** Survival curves in immunocompetent mouse model; **D** Body weight curve combined with immunocompetent mouse model. **E**, **F** Liver and kidney function index were also normal. **G** IHC results suggest the increase of CD8+ cells in both I-DXd/PD-1 monotherapy and the Combo group. **H** The flow cytometric images of a percentage of CD8+ and CD4 + T cells in various treatment groups. **I**, **J** Quantitative comparison of h-graph. **K** Quantitative comparison of CD4+/ CD8 + T cells in various treatment groups. The error bars indicate SEM. Statistical significance was calculated by one-way ANOVA with Tukey’s multiple comparison test. Kaplan–Meier survival (**C**) using the Gehan–Wilcoxon test for the mouse survival data, followed by Bonferroni’s correction for multiple comparisons. ^*^P < 0.05, ^**^P < 0.01, ^***^P < 0.001, ^****^P < 0.0001, ns not significant. IHC images, scale bars, 400 µm and 50 µm, respectively.

We next evaluated the synergistic effects of I-DXd in combination with PD1 immunotherapy in an immunocompetent murine tumor model. An immunocompetent Balb/c mouse model was established by using murine colorectal carcinoma CT26 cells stably expressing human ICAM1 (CT26-hICAM1) due to low tumor formation rates of mouse cervical cancer cell lines. I-DXd, anti-mouse PD-1 monoclonal antibody, or their combination were intravenously administered in comparison with the PBS group (Fig. 6a). Our results show that I-DXd or PD-1 monotherapy yielded moderate tumor inhibitory activities whereas the combo group exhibited the highest antitumor effect, significantly better than I-DXd or PD-1 monotherapy (Fig. 6b). We further evaluated the prognosis of I-DXd and PD-1 combination therapy via Kaplan-Mier analysis. Consistently, I-DXd and Combo groups demonstrated the most favorable prognosis with 83.3% (5/6) mice surviving. In comparison, PBS and PD-1 monotherapy groups had no survivors at the end of the experiment (Fig. 6c). Considering I-DXd and PD-1 combination therapy may induce additive toxicities of each agent, in addition to bodyweight measurement (Fig. 6d), we also performed biochemistry analyses on mouse blood samples collected from immunocompetent Balb/C mice receiving I-DXd/PD-1 combination therapy. As shown in Fig. 6e, f, no obvious changes were observed in either I-DXd/PD-1 monotherapy or the Combo group. Given adaptive immunity is pivotal in PD-1 immunotherapy, we investigated the impact of I-DXd and PD-1 combination therapy on CD4/CD8 + T cell recruitment by IHC staining and flow cytometry (Fig. 6g, h). We observed significant increases in CD8 + T cells and decreases in CD4 + T cells in I-DXd, PD-1, and the Combo group in comparison with the PBS group (Fig. 6i–k). Collectively, our findings support that I-DXd works in synergy with PD-1 immunotherapy via type I IFN responses and subsequently improved CD4/CD8 T cell ratios in TME.

### Non-invasive imaging of ICAM1+ cervical tumor by MRI

**Fig. 7:**
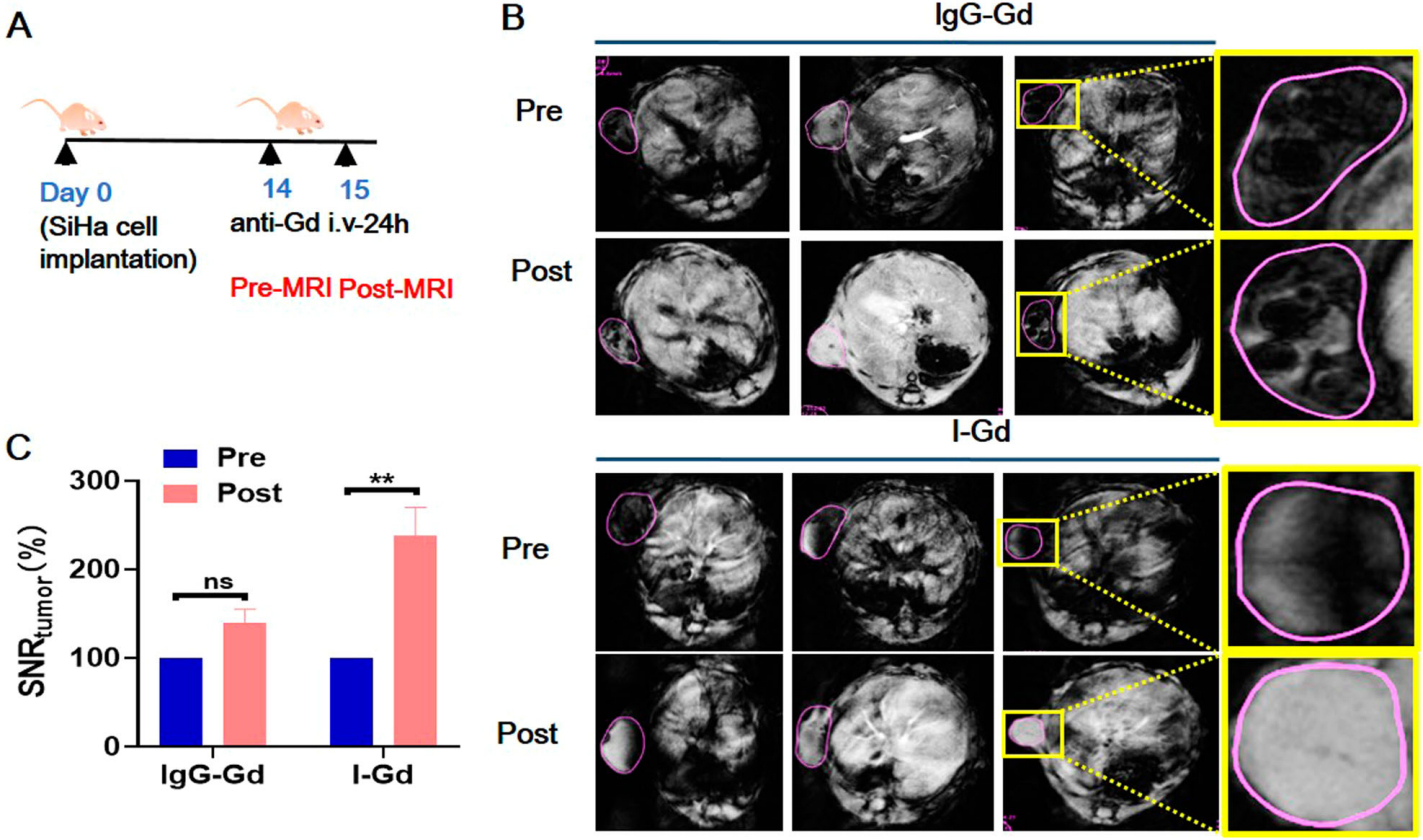
ICAM1-Gd MRI probe non-invasively and quantitatively detects ICAM1 target expression in cervical tumors. **A** Establishment of a mouse model for MRI-based molecular imaging. **B** MRI images of signal intensity changes of the tumor before and after I-Gd treatment (*n* = 4 or 5 per group). **C** Quantization of MRI-T1 signal. The error bars indicate SEM. Statistical significance in (**C**) was calculated by unpaired *t*-test for the mice in two groups. ^*^P < 0.05, ^**^P < 0.01, ^***^P < 0.001, ^****^P < 0.0001, ns not significant.

Given ADC efficacy directly relies on the levels of tumoral antigen expression, tissue biopsy via vaginal endoscopy is frequently required before ADC treatment to assess target adequacy in clinical practice. However, this approach is invasive and potentially causes bleeding and increases the risk of metastasis [44–46]. We previously reported an MRI-based molecular probe by conjugating ICAM1 antibody conjugated with a magnetic resonance T1 contrast agent Gadolinium (I-Gd) [47, 48]. Here we utilized I-Gd to non-invasively quantify tumoral antigen expression of ICAM1 in the CDX model of human cervical tumor (SiHa) via MRI. At pre- and post-I-Gd injections, we performed T1-weighted MR imaging to quantify MRI signal changes at tumor regions as a function of Gd accumulation from administered probes (Fig. 7a, b). In comparison with the non-targeting IgG-Gd group, I-Gd yielded a 138% significantly greater increase in MRI signal in comparison with its background, while IgG-Gd only resulted in a 38% increase (Fig. 7c). The results indicate that the I-Gd MRI probe can accurately and noninvasively detect ICAM1-positive cervical tumors, providing a simple and noninvasive approach to screen patients with high ICAM1 expression and identify those who would benefit from ICAM1-ADC therapy. These MRI signal changes positively correlate with the level of tumoral antigen expression, which could be utilized to non-invasively identify ICAM1+ patients who might benefit from our ADC therapy.

## Discussion

Given the complexity of the TME and its role in immunosuppression, patients with advanced or metastatic solid tumors, including cervical cancer, often encounter significant challenges in treatment, especially due to the scarcity of targeted therapeutic options like ADCs [1, 8, 9]. To address this challenge, our research endeavors to develop novel ADC therapeutics for reprogramming TME, with a specific focus on their effects on innate and adaptive immune cell dynamics. Concurrently, we explore the synergy between ADC treatments and PD-1 immune checkpoint inhibitors, aiming to uncover novel therapeutic avenues.

This study provides the inaugural experimental support for ICAM1 as a viable ADC target in cervical cancer. By employing an unbiased and quantitative approach, we identified ICAM1 as a membrane protein that is overexpressed in cervical cancer cells but not in adjacent normal cervical epithelia. Further analysis, including data from clinical specimens and the GEPIA database, indicated a correlation between ICAM1 overexpression and adverse clinical outcomes in cervical cancer patients, underscoring its potential as a therapeutic target. Importantly, our findings regarding the endocytosis efficiency and antibody binding to ICAM1-expressing cells underline the therapeutic relevance of ICAM1-targeted ADCs. Moreover, ICAM1’s involvement in TME dynamics and pathogenesis further validates its utility as a target for ADC development [49].

Our investigation has yielded two promising ICAM1-targeted ADCs (I-DXd and I-MMAE) that have demonstrated preclinical efficacy and safety in models of cervical cancer. Our comparative studies highlight the superior tumor-cell targeting and elimination capabilities of ICAM1-ADCs over conventional chemotherapy agents like Cis/Pac, in both in vitro and in vivo settings. The design of these ADCs, incorporating enzyme-cleavable linkers (VC or GGFG) and potent cytotoxic agents (MMAE or DXd), achieves an optimal balance between therapeutic efficacy and toxicity [50–52]. Notably, the GGFG-DXd linker used in I-DXd demonstrates advantages in the controlled release of payloads [53, 54], tumor penetration, and bystander-killing effects [55].

I-DXd, in particular, possesses the dual capability of direct cancer cell ablation through antigen recognition and the ancillary eradication of protumor VEGFA+ neutrophils (TANs), which contributes to a more immunologically favorable TME. Moreover, the successful development of I-DXd complements the limitations observed with the only other ADC for cervical cancer, Tisotumab Vedotin, which targets tissue factor (TF) but is associated with significant ocular and peripheral neurotoxicity [56, 57]. I-DXd’s less cytotoxic payloads suggest a reduced risk of these adverse effects. In addition, our current data indicate that most serum markers, including ALT, AST, ALP, CREA, UA, CHE, and TBIL, remained within the normal range and did not show statistically significant differences compared to the PBS control group. Only a slight increase in UREA was observed, suggesting minimal renal impact under the current dosing regimen. We acknowledge that extended treatment duration or additional dosing could increase the risk of cumulative toxicity. If treatment were prolonged, it would be essential to monitor these markers over time to assess for any progressive trends. Increased frequency or duration of administration could potentially lead to elevated levels of UREA or other serum markers, reflecting stress on renal or hepatic systems. Our current data, however, support that the single-agent ADC treatments remain within a safe range under the dosing schedule used. Meanwhile, the potential of combining I-DXd with PD-1 inhibition warrants exploration in clinical settings, as preliminary evidence suggests this combination can enhance CD8 + T cell recruitment, tumor suppression, and overall survival outcomes.

Transcriptomic analysis post-treatment with I-DXd reveals significant modulation of the TME (Figs. 4 and 6), including downregulation of neutrophil activation and upregulation of type I interferon signaling pathways, suggesting a comprehensive improvement in the immune landscape of treated tumors. These findings, coupled with the observed efficacy of I-DXd in combination with PD-1 blockade, underscore the therapeutic potential of targeting ICAM1 in cervical cancer, particularly in TMEs characterized by high mutational burdens and suppressed immune responses [58–61]. Furthermore, the most common adverse effect associated with ADC is peripheral neutropenia caused by off-tumor toxicity of ADC [56, 57]. Our findings suggested that the use of granulocyte colony-stimulating factor (G-CSF) to manage ADC-induced neutropenia [57, 59], while standard, may paradoxically enhance TAN infiltration and attenuate the antitumor efficacy of ADCs. Our data demonstrate that I-DXd selectively eliminates pro-tumor VEGFA+ TANs with high CSF3 expression within the TME, mitigating the potential counterproductive effects of G-CSF and enhancing the immunomodulatory impact of ADC therapy. Through extensive preclinical evaluation, we provide a mechanistic rationale for the combination of ADC and PD-1 inhibition in improving the TME and advancing the treatment of cervical cancer.

In summary, we address the therapeutic challenge of immunosuppression within the TME of advanced cervical cancer by the development of novel ADCs aimed at reprogramming the TME, with I-DXd showing particular promise due to its dual action on cancer cell ablation and pro-tumor VEGFA + TAN eradication, fostering an immunologically favorable TME. The promising synergy of I-DXd with PD-1 inhibition, suggested by enhanced CD8 + T cell recruitment and tumor suppression, alongside a comprehensive modulation of the TME, underscores the potential of ICAM1-targeted therapy to significantly advance cervical cancer treatment.

## Methods

### Cell lines and culture

Human cervical cancer cell lines (SiHa, CaSki, and ME180) were obtained from the cell bank of Zhejiang Cancer Hospital and Shanghai Cancer Hospital, and normal cervical cells (HcerEpic) were purchased from Tongpai (Shanghai) Biotechnology Co., Ltd. Mouse colorectal cell line CT26 were purchased from China Center for Type Culture Collection (Beijing, China). Normal human embryonic kidney HEK293T cell line was purchased from the American Type Culture Collection (Manassas, VA, USA). The culture medium MEM, RPMI-1640, and fetal bovine serum were purchased from Gibco Company. All cells were cultured according to the recommended conditions. Medium containing 10% fetal bovine serum, and 1% penicillin/streptomycin at 37 °C, and 5% CO_2_ incubator. The culture medium was changed once every 2–3 days, and the cells with a cell growth density of about 90% and in the logarithmic growth phase were digested with trypsin for passage and follow-up experiments.

### Antibody libraries and reagents

PE anti-human CD9, Cat: 312106; PE anti-mouse/human CD15 (SSEA-1), Cat: 125606; PE anti-human CD29, Cat: 303004; PE anti-human CD31, Cat: 303106; PE anti-human CD44, Cat: 397504; PE anti-human CD46, Cat: 352402; PE anti-human CD47, Cat: 323108; PE anti-human CD49a, Cat: 328304; PE anti-human CD49b, Cat: 359308; PE anti-human CD49c (integrin α3), Cat: 343803; PE anti-human CD49e, Cat: 328010; PE anti-human CD50 (ICAM-3), Cat: 330005; PE anti-human CD51/61, Cat:304406; PE anti-human CD56 (NCAM), Cat: 362508; PE anti-human CD62E, Cat: 336008; PE anti-human CD62P (P-Selectin), Cat: 304906; PE anti-human CD66a/c/e, Cat: 342304; PE anti-human CD70, Cat: 355104;PE anti-human CD71, Cat: 334106; PE anti-human CD73 (Ecto-5’-nucleotidase), Cat: 344004; PE anti-human CD90 (Thy1), Cat:328110; PE anti-human CD105, Cat: 323206; PE anti-human CD106, Cat: 305806;PE anti-human CD107a (LAMP-1), Cat: 328608; PE anti-human CD117 (c-kit), Cat: 375206; PE anti-human CD126 (IL-6Rα), Cat: 352804; PE anti-human CD140a (PDGFRα), Cat: 323506; PE anti-human CD140b (PDGFRβ), Cat: 323606; PE anti-human CD141 (Thrombomodulin), Cat:344104; PE anti-human CD142, Cat: 365204; PE anti-human CD144 (VE-Cadherin), Cat: 348506; PE anti-human CD146, Cat: 361006; PE anti-human CD152 (CTLA-4), Cat: 349906; PE anti-human CD166, Cat: 343904; PE anti-human CD171 (L1CAM), Cat: 371604; PE anti-human CD181 (CXCR1) Cat: 320608; PE anti-human CD184 (CXCR4) Cat: 306506; PE anti-human CD192 (CCR2) Cat:357206; PE anti-human CD195 (CCR5) Phospho (Ser349) Cat:321606; PE anti-human CD202b (Tie2/Tek) Cat:334206; PE anti-human CD203c (E-NPP3) Cat:324606; PE anti-human CD221 (IGF-1R) Cat:351806; PE anti-human CD227 (MUC-1) Cat:355604; PE anti-human CD254 (TRANCE, RANKL) Cat:347504; PE anti-human CD257 (BAFF, BLYS) Cat:366506; PE anti-human CD266 (Fn14, TWEAK Receptor) Cat:314004; PE anti-human CD271 (NGFR) Cat:345106; PE anti-human CD274 (B7-H1, PD-L1) Cat:329706; PE anti-human CD276 (B7-H3) Cat:351004; PE anti-human CD304 (Neuropilin-1) Cat:354504; PE anti-human CD309 (VEGFR2) Cat:393004; PE anti-mouse CD317 (BST2, PDCA-1) Cat:127104; PE anti-human CD324 (E-Cadherin) Cat:324106; PE anti-human CD325 (N-Cadherin) Cat:350805; PE anti-human CD326 (Ep-CAM) Cat:324206;PE anti-human CD340 (erbB2/HER-2) Cat:324406; PE anti-human CD371 (CLEC12A) Cat: 353604; PE anti-human EGFR Cat:352904; PE anti-human EphA2 Cat: 356804; PE anti-human ROR1 Cat: 357804; PE anti-human VEGFR-3 (FLT-4) Cat: 356204; PE anti-FOLR1 (Folate Binding Protein) Cat: 908304; PE anti-human erbB3/HER-3 Cat:324706; PE anti-human Notch 3 Cat: 345406; PE anti-human PSMA (FOLH1) Cat: 342504; PE anti-human/mouse SSEA-3 Cat: 330312; PE anti-human SSEA-4 Cat: 330406; PE anti-human SSEA-5 Cat: 355204; PE anti-human TM4SF Cat: 20367204; PE anti-human CD54 Cat: 353106; Antibody libraries and reagents are used to screen cancer target candidates using FACS mothods.

Other relative antibodies, PE mouse IgG1 (Cat: 400114), purified anti-human CD54 Antibody (Cat: 322702), and PE anti-mouse IgG1 Antibody (Cat: 406608) were purchased from BioLegend.

### IHC methods

Survival cervical cancer tissue chip (Cat: HUteS136Su01, Lot: XT17-039) was purchased from Shanghai Core Super Company. IHC staining was used to detect the difference in ICAM1 target protein expression in 25 groups of human cervical cancer tissues, paracancerous tissues, and 86 cases of cervical tissues. The expression of ICAM1 (anti-ICAM1 antibody, ab1846317, Sigma; 1:1000) was analyzed semi-quantitatively by tissue microarray. The individual tissues were scored by a pathologist from Zhejiang Cancer Hospital, with no knowledge of sample identity, based on both the intensity of staining and the proportion of positive cells within the section. The IHC staining scoring criteria of ICAM1 are defined as shown in Fig. S2 and entailed a reference to the HER2 target and a differentiation of the positive areas with a tan depth (0, 1+, 2+, 3+). The method of ICAM1-H-Score scoring is detailed as follows: the staining intensity (0, 1+, 2+, 3+) is multiplied by the percentage of positive cells in all tumor cells, and the resulting product represents the final score. The ICAM1-H-Score score results are divided into two categories: ICAM1+ (Positive) and ICAM1-(negative). The ICAM1+ (positive) category is further subdivided into three expression levels: The ICAM1-H-Score can be classified as low (1–10), moderate (10–40), or high (40–300). Conversely, ICAM1-(negative) is defined as negative (0), which indicates the complete absence of the specific ICAM1 expression. To illustrate, the staining intensity is 2+, the percentage of positive tumor cells is 15%, and the calculated ICAM1-H-Score is Moderate (30), which is determined as ICAM1 + (Positive). Anti-Ly6G antibody (Cat: ab238132), anti-MPO antibody (Cat: ab208670), CD8α (D4W2Z) XP®Rabbit mab (Cat: 98941), anti-CD4 antibody (Cat: ab288724) are used for IHC staining in animal models.

### Flow cytometry flow cytometry

Quantification of ICAM1 expression in cells: Flow cytometry (Beckman Coulter, CytoFLEX LX) was used to evaluate the expression of ICAM1 on the surface of cervical cancer cells and normal cells. Quantitative analysis of cellular antigen expression was determined with Quantum TM Simply Cellular® microspheres (Bangs Laboratories) by using a method provided by the manufacturer. One million cells were harvested from each cell line and rinsed twice with PBS. The harvested cells were blocked with 1% bovine serum albumin (BSA) in PBS for 30 min at 4 °C. After BSA blocking, the cells were incubated with PE anti-human CD54 (Cat: 353106) for 1 h at 37 °C. PE anti-mouse IgG1 (Cat: 406608) was used as a control. The cells were washed three times with 1% BSA in PBS, then resuspended in PBS, and the expression intensity of ICAM1 in each cell line was evaluated by flow cytometry. The PE anti-mouse IgG1 (Cat: 406608) and Purified anti-human CD54 (Cat: 322702) are purchased from BioLegend Company.

Multi-colored flow cytometry: Fixable Viability Stain 780(Cat: 565388V500); Rat Anti-Mouse CD45(30-F11)(Cat: 561487); FITC Rat Anti-CD11b(M1/70) (Cat: 557396); BV421 Hamster Anti-Mouse CD3e(145-2C11)(Cat: 562600); APC Rat Anti-Mouse CD4(RM4-5) (Cat: 553051); PE-Cy7 Rat Anti-Mouse CD8a(53-6.7) (Cat: 552877); Purified Rat Anti-Mouse CD16/CD32 (Mouse BD Fc Block)(2.4G2) (Cat: 553141); Those antibodies Antibodies are used in multi-colored flow cytometry to detect changes in the ratio and number of CD4/CD8 + T cells.

### Cell viability

Viability assessment of drug killing: Three to five thousand cells were plated into 96-well plates and incubated with various concentrations of drugs. After 72 h, CCK8 reagents were added and incubated for 1–4 h at 37 °C. Cell viability was examined by the CCK8 assay and detected absorption values at 450 nm.

Viability assessment of ICAM1 antibody: Ten thousand SiHa cells were plated into 96-well plates and incubated with purified anti-human CD54 antibody (Cat: 322702) at the indicated time points (0-30-60-120-240 min). Cell viability was examined by the CCK8 assay and detected absorption values at 450 nm.

### Determination of internalization

The subcellular location of the target protein was observed in different cell lines. One million cells were inoculated in confocal plates, and cultured overnight at 37 °C. The cells were washed in PBS and blocked in 1% BSA for 30 min at 4 °C, and incubated with fluorescent-labeled antibodies or drugs for 1 h. After staining with Hoechst, they were imaged with a confocal fluorescence microscope (NIKON, A1 HD25). Cy3-NHS (Cat: R-H-6551), ICAM1-MMAE and ICAM1-MMAE are used to synthesize ICAM1-MMAE-cy3 and ICAM1-DXD-cy3. Mouse tumor infiltrating tissue neutrophil separation solution kit (Cat: P2430) and mouse peripheral blood neutrophil separation kit (Cat: P9201) were used to isolate TAN and peripheral blood neutrophils.

The confocal imaging was used to determine whether ICAM1-ADCs could selectively enter cervical cancer cells and quickly transport them to intracellular lysosomes. The cells were inoculated, cultured overnight at 37 °C, and incubated with ICAM1 primary antibody with 0 min, 30 min, 60 min, 120 min and 240 min at 37 °C. Then all cells were fixed with 4% paraformaldehyde, stained with Hoechst, and imaged with a confocal instrument. The internalization efficiency was calculated by an established formula [62]: internalized% = (0 min MFI −t/min/MFI)/0 min × 100%. Purified anti-human CD54 antibody (Cat: 322702); PE-conjugated rat anti-mouse IgG (BioLegend, San Diego, CA).

Co-localization of ICAM1 antibody and lysosome were assessed by confocal fluorescent imaging. The cells were inoculated, incubated with ICAM1 primary antibody, fixation. Then permeabilization, and blocking were respectively performed by 4% formaldehyde, 0.5% TrotonX-100, and 0.5% BSA. The cells were incubated with LAMP1 antibody at 4 °C overnight and then with cy5 anti-mouse lgG secondary Antibody for 2 h at room temperature. After staining Hoechst, confocal images were captured by a NIKON A1 HD25 microscope.

### Synthesis and characterization of preparation and characterization of ADCs

Monoclonal ICAM1 chimeric antibodies were covalently conjugated with two different linkers (MC-VC-PAB and MC-GGFG) and two different warheads (MMAE and DXd) to create a group of ICAM1-ADCs, which includes ICAM1-MC-VC-PAB-MMAE (I-MMAE) and ICAM1-MC-GGFG-DXd (I-DXd). MMAE and DXd are commonly used warheads in clinical ADCs. The DAR values of two types of ICAM1 ADCs were determined using hydrophobic interaction chromatography (HIC), ensuring their optimal efficacy and toxicity.

#### In Vitro cytotoxicity studies

Human cervical cancer cell lines (SiHa, CaSki) and normal cells (HcerEpic, 293T) were tested using the CCK8 kit (Cat: BS350B) from Bioshrp Company. The cells were inoculated at a density of 3000 cells per well in 96-well petri dishes and cultured overnight. ICAM1-ADCs (I-MMAE, I-DXd), an ICAM1 monoclonal antibody, and two chemotherapy drugs (Cis, Pac), were added to the wells at drug concentrations ranging from 0 μg/ml to 10 μg/ml. The addition was carried out with 10-fold continuous dilution, resulting in a total of eight concentrations and three parallel wells per drug. After culturing the cells and drugs for 96 h, discard the original culture medium. Dilute the CCK8 detection reagent ten times with fresh cell culture medium and add 100 PL/well to the 96-well plate. Incubate the plate at 37 °C for 1–4 h, and measure the absorbance (OD) at the absorption wavelength of 450 nm.

### Animal experiments

All animal studies were performed according to the protocols approved by the Institutional Animal Care and Use Committee (IACUC) of Zhejiang Cancer Hospital. The in vivo therapeutic efficacy of ICAM1-ADCs was evaluated in both standard and late-stage cervical tumor models. 4 × 10^6^ SiHa cells suspended in 100 μl PBS (contained 50% Matrigel) were injected subcutaneously into the right flank of 4-6-week-old female nude mice. When the tumor volume reached 50 to 80 mm^3^(standard model) or approximately 500 mm^3^ (late-stage model), the mice were randomized into 5 groups (*n* = 6 or 7 per group) and dealt with different treatments of PBS, Cis, ICAM1 monoclonal antibody (I Ab), I-DXd or I-MMAE in an equivalent dosage of 5mg/kg with QW × 2 manner via tail vein injection. In addition, for the combination therapy study, 6 × 10^6^ CT26-hICAM1 cells suspended in 100 μL PBS (contained 50% Matrigel) were injected subcutaneously into the right flank of 4–6-week-old female BALB/c mice. When the tumor volume reached 300 mm^3^, BALB/c mice were randomized into 4 groups of 7 and treated with PBS, 10 mg/kg anti-mouse PD-1 antibody (InVivoPlus, Cat: BP0146) for Q3D × 2, 5 mg/kg I-DXd for Q3D × 2, and combination with I-DXd and PD-1. Tumor sizes and body weight were recorded every other day or daily. The long diameter (a) and short diameter (b) of tumor nodules were measured with calipers and the tumor volume was calculated according to the tumor volume: V = a×b^2^/2. The tumor-bearing mice were recorded and euthanized at the end of the study, or when tumors reached 1500 mm^3^.

At the end of the experiments, blood serum was collected from each experimental group (*n* = 2–5 per group). The serum levels of alanine aminotransferase (ALT), aspartate aminotransferase (AST), alkaline phosphatase (ALP), total bilirubin (TB), blood concentration of creatinine (BUN) and creatinine (Cre) and other liver, kidney and inflammation indicators were determined with biochemical analyzer Hitachi 7180 (Hitachi, Yokohama).

### Western blot analysis

Isolation of TANs and peripheral blood neutrophils: Mouse tumors infiltrating tissue neutrophil separation solution kit (Cat: P2430) and mouse peripheral blood neutrophil separation kit (Cat: P9201) were used to isolate TANs and peripheral blood neutrophils. Peripheral blood and tumor tissues were collected from three tumor-bearing female nude mice at the same time. The tumor tissue was minced and ground into a cell suspension and then filtered through a 70 μm filter membrane to form a single cell suspension. According to the manufacturer's instructions, the target cells were isolated and collected for further use. TANs, peripheral blood neutrophils, and mouse cervical cancer cells (U14-ICAM1) were lysed by SDS lysis buffer supplemented with Phosphatase Inhibitor Cocktail. All samples were analyzed by SDS-PAGE and then transferred to PVDF membranes. The membranes were blocked with 5% bovine serum albumin (Cat: D8340) for 1 h at room temperature and then incubated with the primary antibody anti-Cathepsin B (Cat: ab214428) while anti-β-Actin (Cat: EM21002) as control at 4°C overnight. Then the membranes were incubated with secondary antibodies for 1h at room temperature. The protein bands were detected using the ECL reagent (Cat: BL523B) and Amersham ImageQuant 800 systems.

### RNA-seq analysis

RNA extraction, cDNA library preparation, RNA sequencing, quality control, and transcriptome profiling of endpoint tumors in different treatment groups (PBS, ICAM1 mAb, I-DXd, and I-MMAE, three repeated samples per group) of late-stage tumor mouse models were performed by Novogene (Beijing, China). The GO enrichment analysis and Reactome pathway analysis enrichment of DEGs were conducted between PBS, Cis, I-DXd and I-MMAE treatment groups. The clinical relevance and prognosis of VEGFA+ TANs in the GSE63514 database and TCGA cohort were assessed in gene set variation analysis (GSVA). In addition, The CIBERSORT and xCELL algorithms were employed to deconvolute bulk RNA-seq profiles and quantify the infiltration of fibroblasts, immune cells (such as CD8+、CD4+ T cells), and other cell types. The changes between high and low TAN-related GSVA score groups in the proportion of immune cells in the TME of cancer patients in the TCGA cohort were assessed in EPIC (https://gfellerlab.shinyapps.io/EPIC_1-1).

### I-Gd preparation and MRI imaging

I-Gd, lgG-Gd drugs prepared: The DTPAA is gradually added to the lgG or ICAM1 antibody, and the reaction is continuously monitored in the two buffers, resulting in the production of lgG-Gd and I-Gd [47]. Briefly, DTPAA (1 mg) was slowly added to lgG or ICAM1 Ab (1 mg) in 1 ml of buffer system 1 (NaHCO_3_ buffer, pH = 9.0), and the solution should be kept in the dark at 37 °C for four hours. The DTPA-conjugated IgG or ICAM1 Ab was transferred to a dialysis bag and placed in a beaker of ultra-pure water for dialysis overnight, with unbound small molecules being filtered out. Subsequently, the liquid within the dialysis bag was subjected to freeze-drying at a temperature of −80 °C, resulting in the formation of a powder. This powder was then dissolved in 1 ml of buffer 2 (sodium citrate buffer, pH = 6.5), and subsequently transferred to a tube containing 0.3 mg of Gadolinium (III) chloride hexahydrate (Cat: G7532-5g) for 24 h rotated at 37 °C. The free Gd^3+^ was removed by Ultra-4 Centrifugal Filter (30 K MWCO), and IgG-Gd or I-Gd was redispersed in PBS and measured the concentration of BCA protein for subsequent use.

MRI imaging: 6-week-old female tumor-bearing (SiHa) nude mice were randomized into two groups (I-Gd and lgG-Gd, n = 4 or 5 per group), when the tumor volume reached 500 to 1000 mm^3^.The target group (I-Gd) and the control group (lgG-Gd) were both administered an equivalent dose of 5 mg/kg via the tail vein, and MRI imaging was performed 24 hours later. The MRI instrument (Bruker BioSpin MRI, 7.0-T) was used to perform T1 and T2-weighted MR imaging to quantify MRI signal changes. MRI gradient echo sequence was given to each group. The following parameters were used: TR/TE = 1300/7.2 ms, 1 mm slice thickness, FOV 71 × 85 mm, and FOV 256 × 256 matrix.

### Quantification and statistical analysis

GraphPad Prism 8.0 and FlowJo V10 were used to analyze the experimental data, and the error bars indicate SEM. Statistical significance was calculated by one-way ANOVA with Tukey’s multiple comparison test. The difference between the two groups was analyzed by *t*-test. Kaplan–Meier survival using the log-rank (Mantel–Cox) test for the clinical survival data or the Gehan-Wilcoxon test for the mouse survival data, followed by Bonferroni’s correction for multiple comparisons. ^*^P < 0.05, ^**^P < 0.01, ^***^P < 0.001, ^****^P < 0.0001, ns, not significant. GraphPad Prism 8.0 is used to draw related pictures of the experimental data. Quantify the signal intensity of the tumor and draw the region of interest (ROI) around the whole tumor at the same imaging depth. The pixel intensity is calculated by ImageJ software and normalized to ROI.

## Supplementary information

Supplementary Figures 1–9 and Captions

## Data Availability

All data in our study are available upon reasonable request.
