# Supplementary material for: Eliminating VEGFA+ tumor-associated neutrophils by antibody-drug conjugates boosts antitumor immunity and potentiates PD-1 immunotherapy in preclinical models of cervical cancer
Source: Cell Death Dis. 2025 Feb 19;16(1):115. doi: 10.1038/s41419-025-07402-9 (PMC11840153; doi:10.1038/s41419-025-07402-9)
Supplement: Supplementary file 1 — Supplementary Figures 1–9 and Captions [file 41419_2025_7402_MOESM1_ESM.docx]

**Eliminating VEGFA+ tumor-associated neutrophils by antibody-drug conjugates boosts antitumor immunity and potentiates PD-1 immunotherapy in preclinical models of cervical cancer**

*Shili Yao^1,2^, Lu Sun^2,3^, Ye Lu^2^, Xiu Zhu^2,3,5^, Rui Xu^2,3,4^, Tong Yang^2^, Huarong Tang^2,3,6*^, Peng Guo^2,3*^, Tao Zhu^2,3,5*^*

1. School of Materials Science and Engineering, Faculty of Medicine, Tianjin University, Tianjin, China
2. Hangzhou Institute of Medicine (HIM), Chinese Academy of Sciences Hangzhou, Zhejiang, China
3. Zhejiang Cancer Hospital, Hangzhou, Zhejiang, China
4. Institute of Molecular Medicine, Hangzhou Institute for Advanced Study (UCAS), Hangzhou, Zhejiang, China
5. Department of Gynecological Oncology, Zhejiang Cancer Hospital, Hangzhou, Zhejiang, China
6. Department of Gynecological Radiotherapy, Zhejiang Cancer Hospital, Hangzhou, Zhejiang, China

*These authors contributed equally: Huarong Tang, Peng Guo, Tao Zhu.

*Corresponding author: Peng Guo, Hangzhou Institute of Medicine (HIM), Chinese Academy of Sciences Hangzhou, Zhejiang 310018, Zhejiang Cancer Hospital, Hangzhou, China. E-mail: [guopeng@ucas.ac.cn](mailto:guopeng@ucas.ac.cn); Huarong Tang, [tanghr@zjcc.org.cn](mailto:tanghr@zjcc.org.cn); and Tao Zhu, [zhutao@zjcc.org.cn](mailto:zhutao@zjcc.org.cn).

**Supplenmentary Fig. 1 The whole tissue sections of ICAM1 staining. A** Representative pictures of tumor tissues in cervical cancer clinical specimens. **B** The representative picture of normal tissue. The red box area is the image represented in Figure 1c. Scale bars, 1.25mm.

**
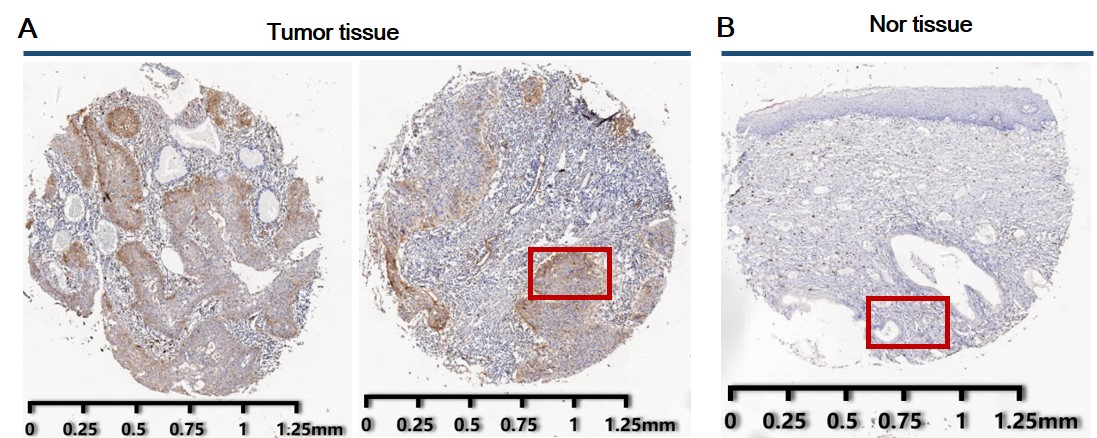
**

**Supplenmentary Fig. 2 Staining intensity grading of ICAM1 IHC.** Representative images of the 4 staining intensities of ICAM1 IHC on human cervical cancer tissue microarray, including negative, low, moderate, and high. IHC images, scale bars, 1.25mm and 200µm, respectively.

**
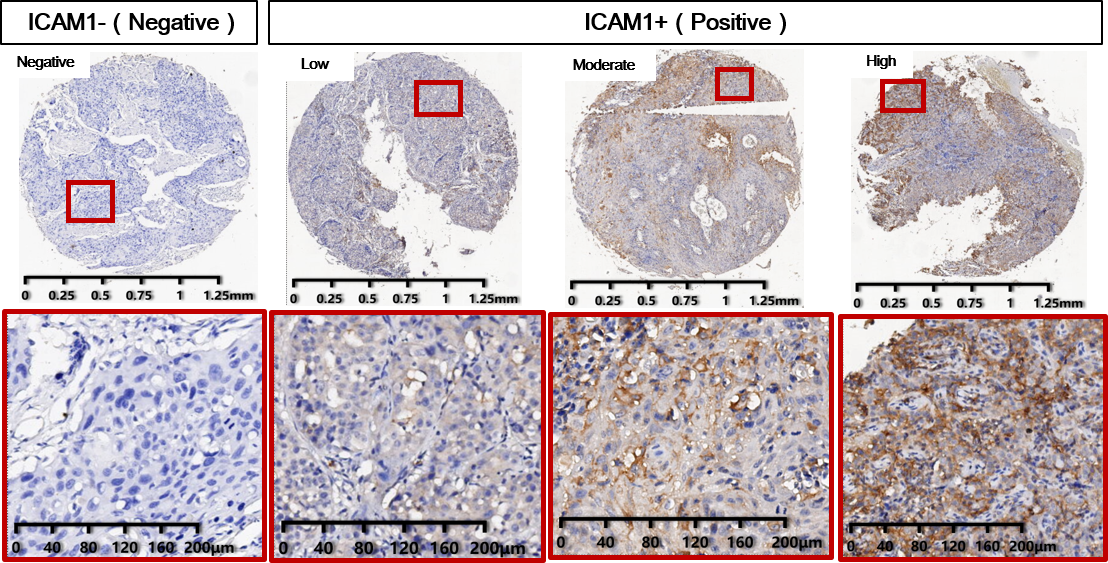
**

**Supplenmentary Fig. 3 Tumor cell viability test during internalization process. A**, **B** The CCK8 assays results of two cervical cancer cells (SiHa and CaSki) after ICAM1 antibody treatment in different time points (0-30-60-120-240 min). The error bars indicate SEM. Statistical significance was calculated by unpaired t-test in two groups. *P < 0.05, **P < 0.01, ***P < 0.001, ****P < 0.0001.

**
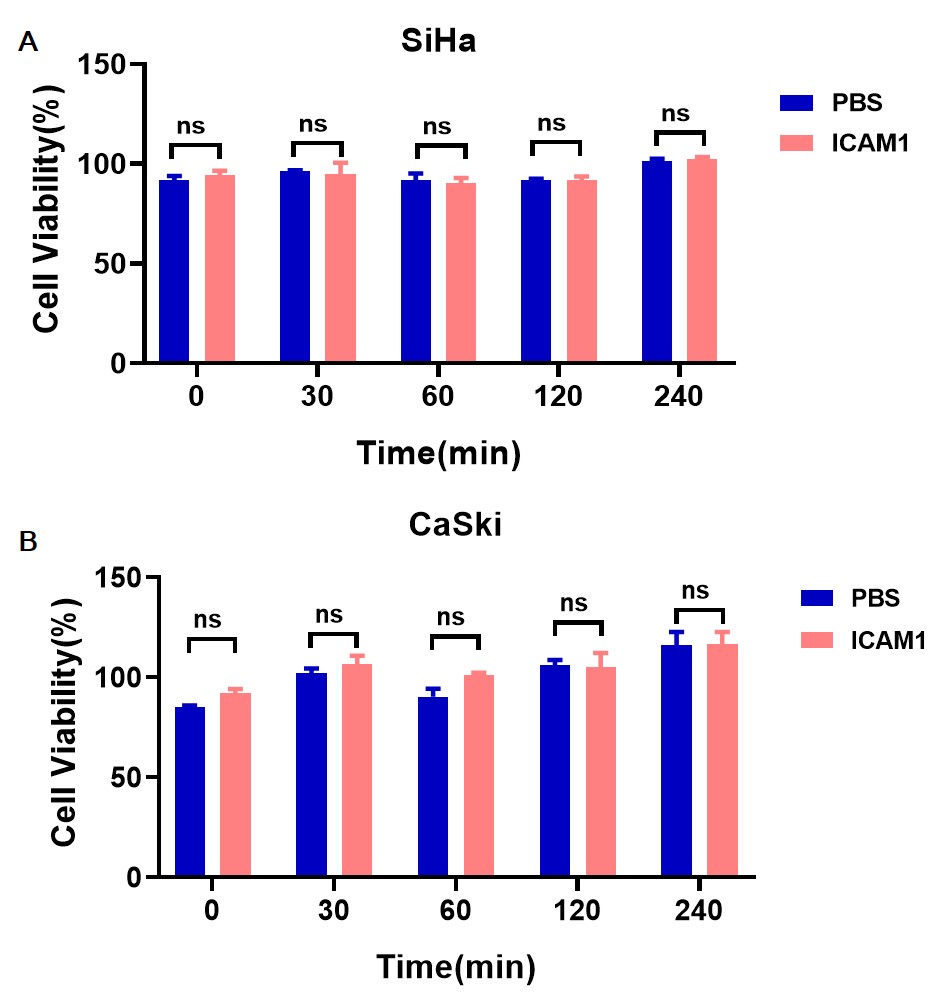
**

**Supplenmentary Fig. 4 The colocalization of internalized ICAM1 antibodies with the lysosome(LAMP+). A**, **B** Representative images of the ICAM1 antibody colocalized with the lysosome of cervical cancer cells (SiHa and CaSki) after internalized 5- and 60- min. Immunofluorescence images, scale bars, 20µm.

**
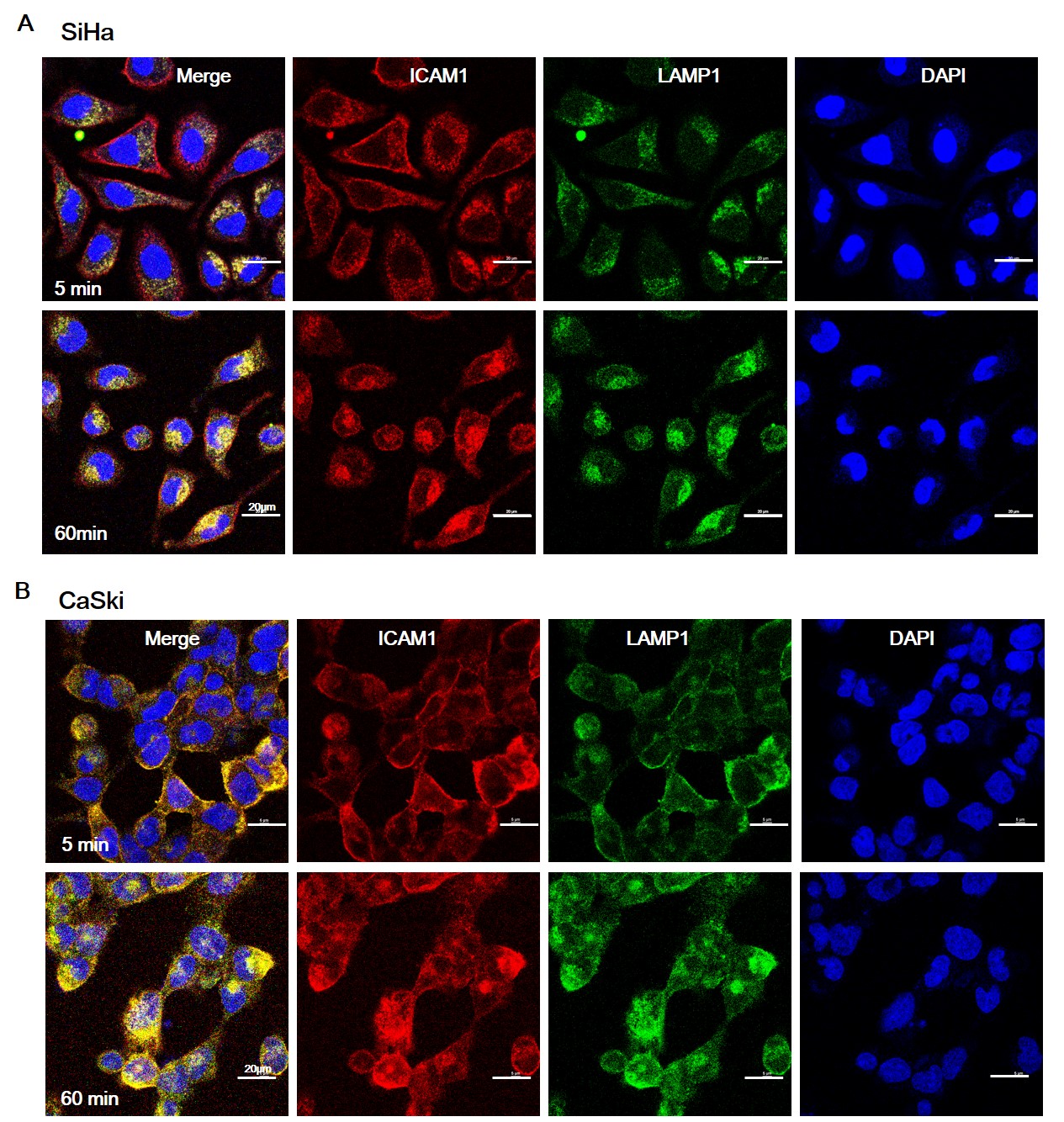
**

**Supplenmentary Fig. 5 ICAM1 ADCs exhibited favorable antitumor activity in vitro.** **A** The killing effects of various drugs on tumor cells (SiHa, CaSki). **B** The killing effects of various drugs on normal cells (293T, HcerEpic).

**
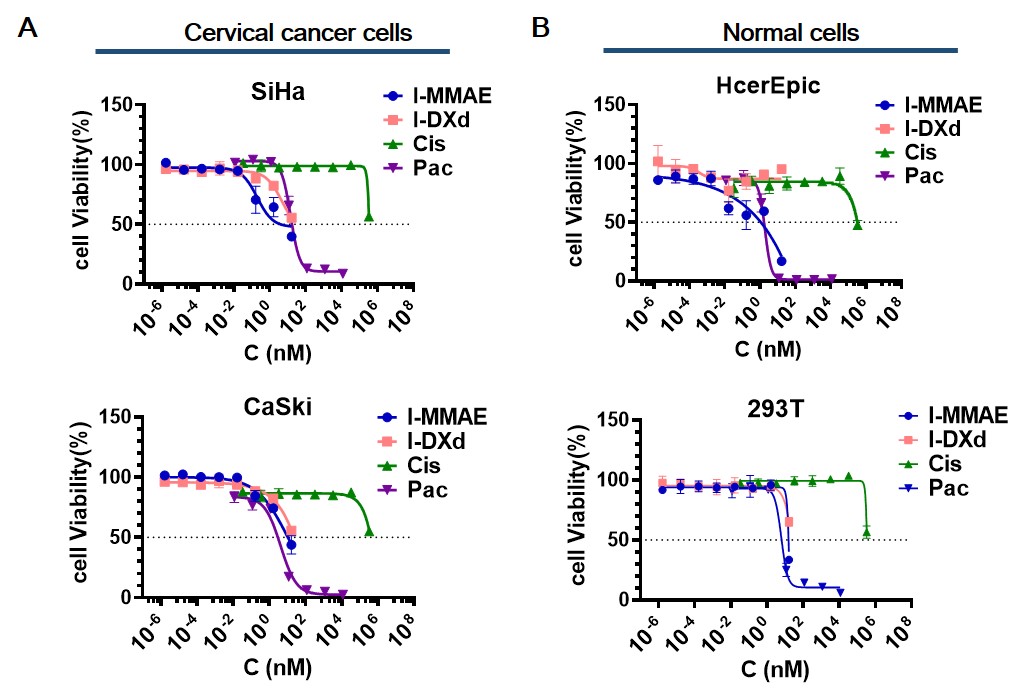
**

**Supplenmentary Fig. 6 The whole tissue sections of Ly6G and MPO staining. A** Representative pictures of Ly6G+ cells in cervical cancer clinical specimens. **B** The representative picture of MPO+ cells. The red box area is the image represented in (Fig. **3C**). Scale bars, 2.5mm.


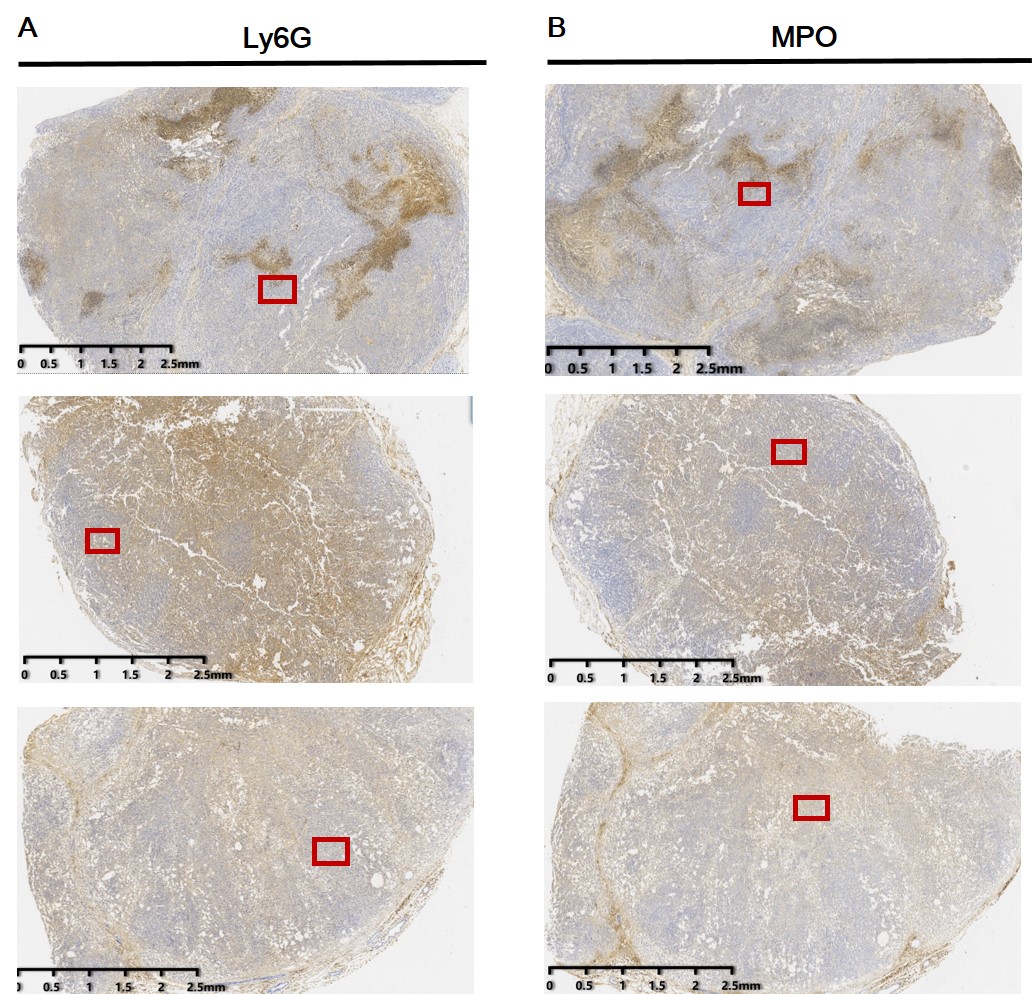


**Supplenmentary Fig. 7 The full length western blot of Figure 4i.** The original immunoblot without cutting is shown below. β-actin was used as the loading control.


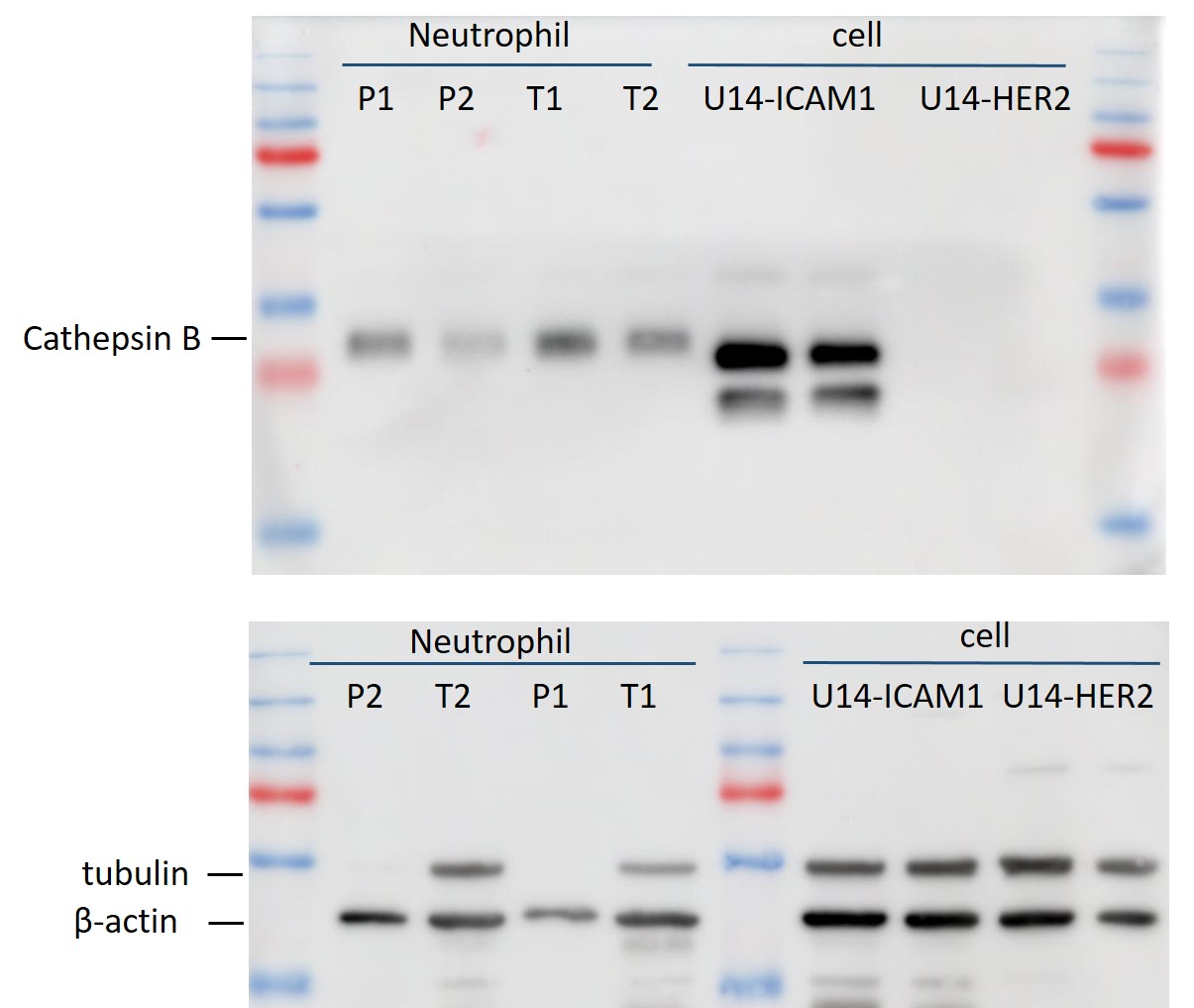


**Supplenmentary Fig. 8 The GO pathways of ICAM1 antibody by antibody-bound ICAM1 using RNA transcriptomic analyses.** The top 20 up- and down-regulated signaling pathways as illustrated.


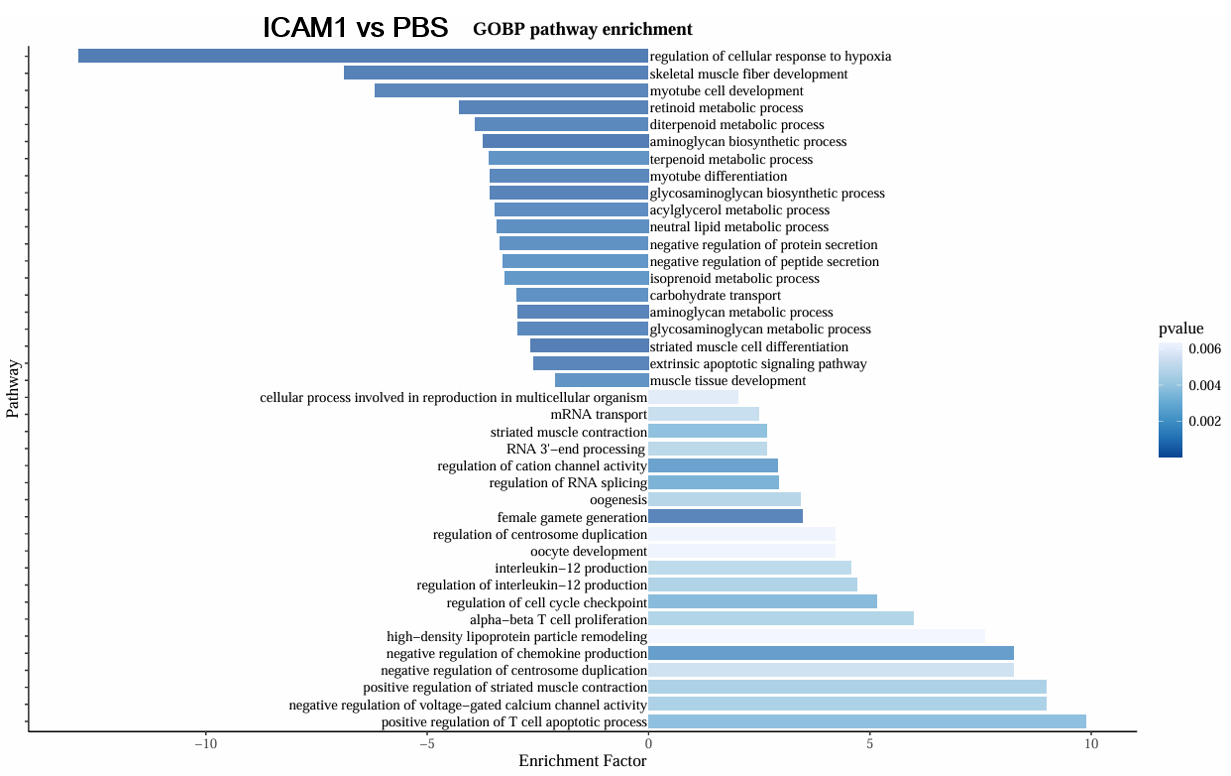


**Supplenmentary Fig. 9 Differential expression of TAN- and IFN-related genes in I-MMAE.** **A**, **B** Protumoral genes related to TAN are slightly downregulated in I-MMAE treatment (n=3 per group). **C**, **D** IFN-related genes are slightly upregulated. The error bars indicate SEM. Statistical significance in (**B** and **D**) was calculated by unpaired t-test for the mouse in two groups. *P < 0.05, **P < 0.01, ***P < 0.001, ****P < 0.0001.

**
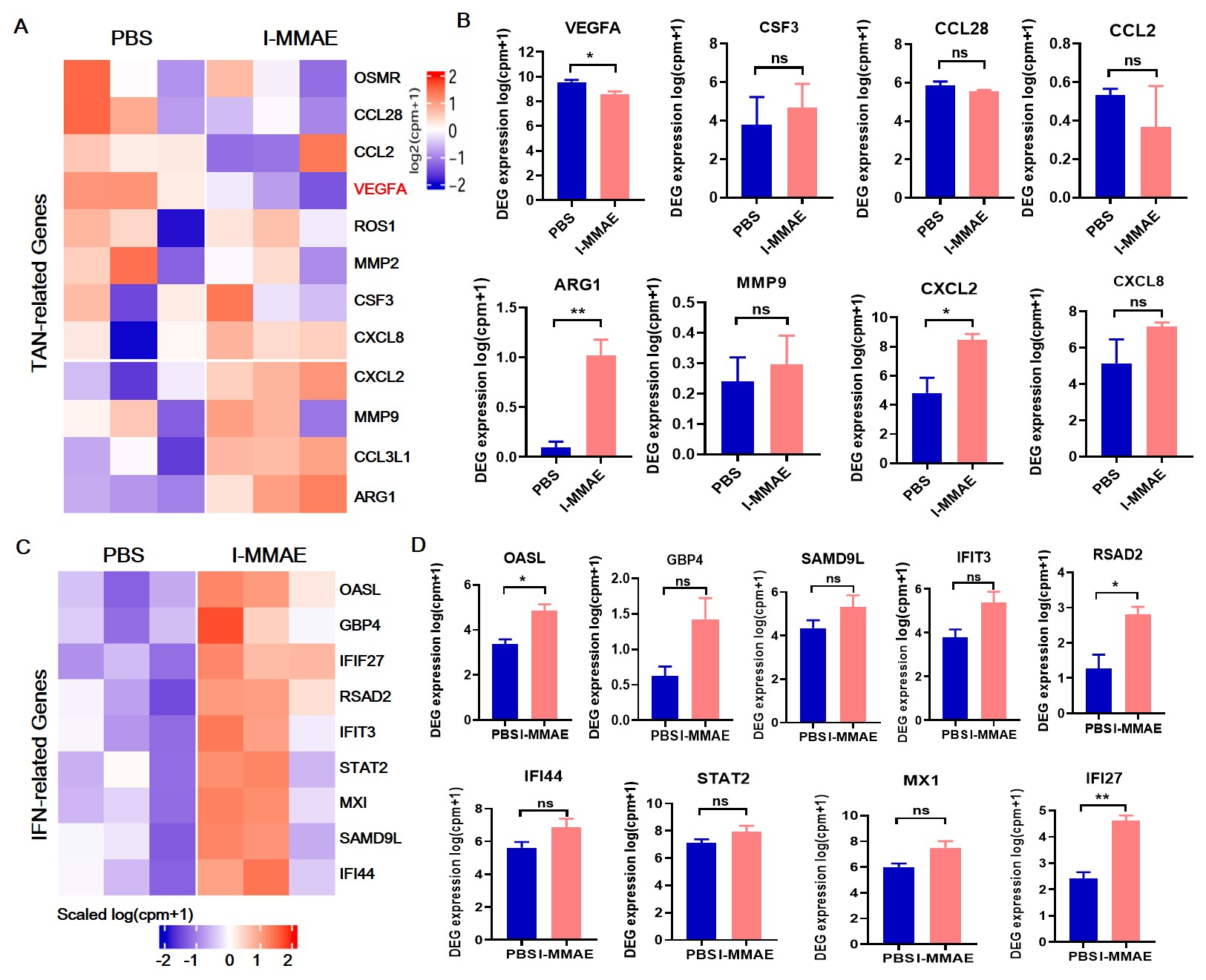
**
